# BRIGHT Enables High‐SNR Live‐Cell Imaging of Non‐Repetitive Sequences via Bivalent Fluorescent Nanobody‐Mediated Cascade‐Dependent Illumination

**DOI:** 10.1002/advs.202513014

**Published:** 2025-10-17

**Authors:** Lei Feng, Tao Huang, Yanxi Han, Meng Tian, Duo Wang, Zilong Mei, Yu Ma, Yingshuo Ma, Jinming Li, Rui Zhang

**Affiliations:** ^1^ National Center for Clinical Laboratories Institute of Geriatric Medicine Chinese Academy of Medical Sciences Beijing Hospital/ National Center of Gerontology Beijing 100730 P. R. China; ^2^ National Center for Clinical Laboratories Chinese Academy of Medical Sciences & Peking Union Medical College Beijing 100730 P. R. China; ^3^ Beijing Engineering Research Center of Laboratory Medicine Beijing Hospital Beijing 100730 P. R. China; ^4^ Peking University Fifth School of Clinical Medicine Beijing 100730 P. R. China; ^5^ College of Life Sciences University of Chinese Academy of Sciences Beijing 101408 P. R. China; ^6^ University of Chinese Academy of Sciences Medical School Beijing 101408 P. R. China

**Keywords:** bivalent fluorescent nanobody, cascade‐dependent illumination, high signal‐to‐noise ratio, live‐cell imaging, non‐repetitive genomic loci

## Abstract

Genomic loci and genome‐independent DNA exhibit heterogeneous dynamics related to physiological function. Live‐cell imaging of non‐repetitive sequences is essential but limited by low signal‐to‐noise ratio (SNR), restricting accurate identification and dynamic tracking. Here, the development of a BRIGHT (bivalent near‐infrared nanobody‐mediated cascade illumination of genomic loci for high‐SNR tracking) system for non‐repetitive sequence live imaging is reported. BRIGHT employs dCas9‐n×ALFA to target genomic loci and realizes high SNR by triggering cascade‐dependent illumination via bivalent binding and antigen‐dependent illumination of a bivalent near‐infrared fluorescent nanobody targeting ALFA tags (Bi‐NIR‐Fb_ALFA_). BRIGHT achieves ≈6.26‐fold and ≈6.76‐fold higher SNR than SunTag and PP7‐PCP in telomere labeling, and enables non‐repetitive DNA imaging with SNR up to 284.84. Furthermore, BRIGHT tracks chromatin dynamics comparably to previous tools, links dynamics and nuclear positioning to transcriptional activity, and detects the distribution and dynamic heterogeneity of extrachromosomal circular DNA (eccDNA). Overall, BRIGHT dramatically improves the SNR of non‐repetitive sequence imaging and offers an innovative tool for studying genomic dynamics and gene regulation.

## Graphical abstract

BRIGHT is a newly developed live‐cell imaging system that integrates cascade amplification with background denoising through the bivalent binding capability and antigen‐dependent stability of a bivalent fluorescent nanobody targeting ALFA peptides. It enables high signal‐to‐noise ratio imaging of non‐repetitive DNA loci (up to 284.84), features a streamlined three‐component design, offers flexible modularity, and demonstrates broad applicability.

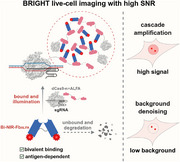

## 1 Introduction

Live‐cell imaging of genomic loci and genome‐independent DNA elements, such as viral DNA and extrachromosomal circular DNA (eccDNA), is crucial for understanding cellular functions and disease mechanisms.^[^ ^1^, ^2^, ^3^ ^]^ Earlier fluorescent tools utilized large integrated DNA arrays (e.g., LacO^[^ ^4^ ^]^) or engineered DNA‐binding proteins (e.g., zinc fingers^[^ ^5^ ^]^), often disrupting native target functions and posing technical challenges.^[^ ^6^ ^]^

Recently, Clustered Regularly Interspaced Short Palindromic Repeats‐deactivated CRISPR‐associated protein 9 (CRISPR‐dCas9) has been adapted for DNA imaging.^[^ ^7^, ^8^ ^]^ Detectable signals usually require 150–200 fluorescent molecules at a given locus,^[^ ^9^ ^]^ prompting the development of various linear signal amplification strategies for labeling repetitive sequences.^[^ ^9^, ^10^, ^11^, ^12^, ^13^, ^14^, ^15^ ^]^ However, imaging of non‐repetitive sequences remains a key goal, as they comprise ≈40–50% of the human genome and are essential for core biological processes.^[^ ^16^ ^]^ While non‐repetitive sequence imaging has been achieved, the signal‐to‐noise ratio (SNR) remains insufficient for accurate tracking.^[^ ^15^, ^17^, ^18^, ^19^, ^20^, ^21^ ^]^ The key to high‐SNR imaging lies in signal amplification and background denoising.^[^ ^6^ ^]^ Signal amplification strategies like CRISPR tiling (e.g., CRISPRdelight^[^ ^20^ ^]^ and Oligo‐LiveFISH^[^ ^21^ ^]^) and strong cascade amplification (e.g., CRISPR FISHer^[^ ^17^ ^]^ and SIMBA^[^ ^18^ ^]^) allow non‐repetitive sequence imaging, but the SNR remains below 30 due to the background from “always‐on” fluorescent proteins (FPs). Background denoising strategies aim to lower the intensity (e.g., CRISPR‐MB^[^ ^22^ ^]^) or the number of unbound fluorophores (eg, fCRISPR^[^ ^23^ ^]^), but imaging non‐repetitive sequences with linear amplification yields limited signal (eg, CRISPR/Pepper‐tDeg^[^ ^19^ ^]^). Therefore, current methods have yet to achieve both strong cascade amplification for high signal and effective background denoising for low noise in non‐repetitive sequence labeling.

**Figure 1:**
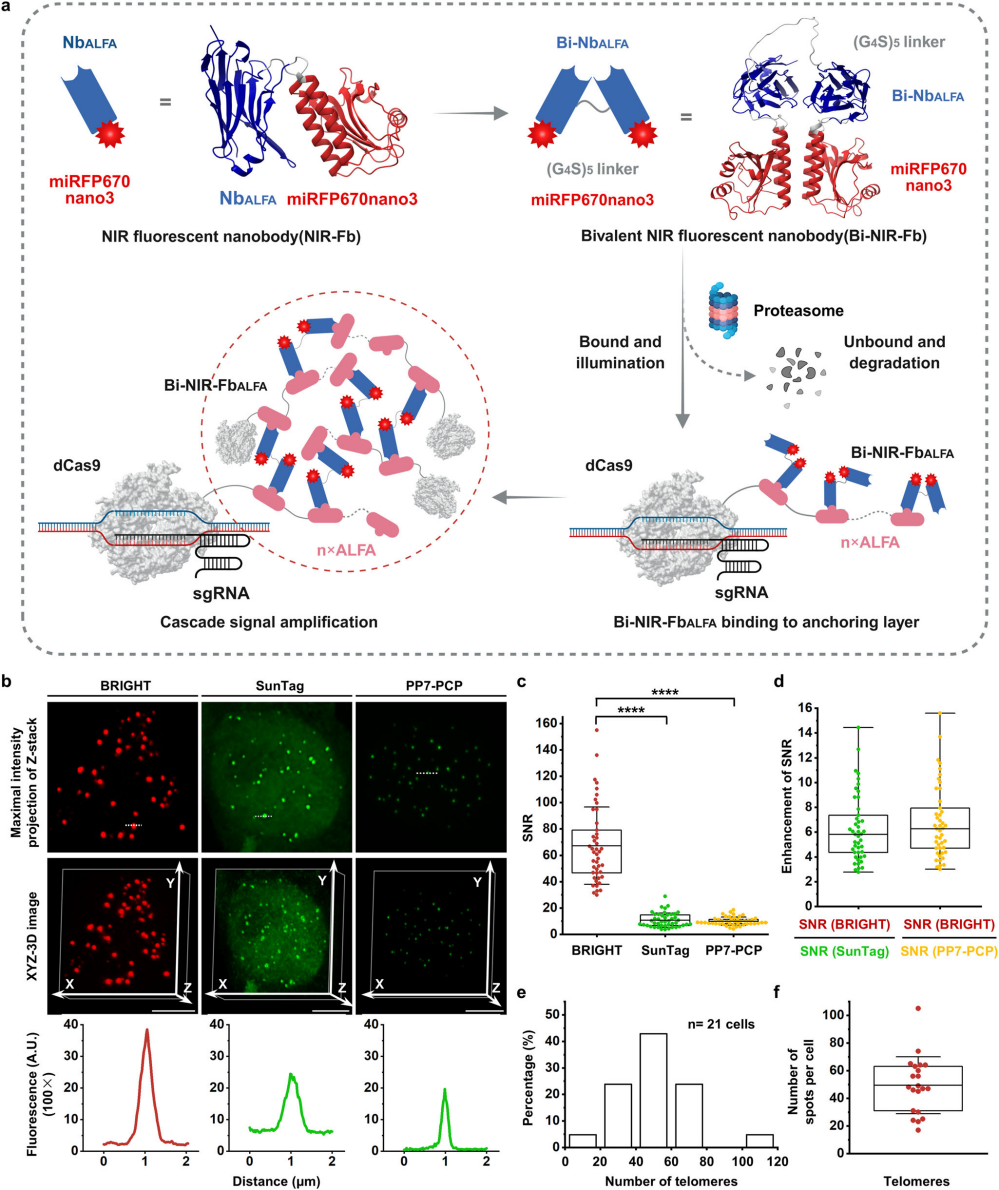
The design of the BRIGHT imaging system and its application in telomere imaging. a) Schematic diagram of the BRIGHT live‐cell imaging system. miRFP670nano3 (red) performs as an internally inserted fluorescent tag at the N65/C66 site of Nb_ALFA_ (blue) to generate a NIR‐Fb_ALFA_. The bivalent construct (Bi‐NIR‐Fb_ALFA_) consists of two NIR‐Fb_ALFA_ units connected by a flexible (G_4_S)_5_ linker (gray), enabling simultaneous recognition of two ALFA tags. Dual ALFA engagement is required to stabilize Bi‐NIR‐Fb_ALFA_, and partial or no binding leads to rapid degradation via the ubiquitin‐proteasome system. To target specific genomic loci and realize cascade signal amplification, dCas9 is fused with tandem ALFA tags (dCas9‐n×ALFA). Upon dCas9‐n×ALFA/sgRNA binding to the target site, the anchoring layer is formed, and the first layer of Bi‐NIR‐Fb_ALFA_ is recruited. The bivalent binding further facilitates the accumulation of free dCas9‐n×ALFA and Bi‐NIR‐Fb_ALFA_ molecules, establishing a cascade signal amplification. Benefited from the antigen‐dependent stabilization, ALFA‐unsaturated Bi‐NIR‐Fb_ALFA_ will be rapidly degraded, ensuring a low nuclear background. The high imaging SNR arises from a strong target‐specific signal and minimal background fluorescence. Protein structures were predicted with AlphaFold3^[^ ^75^ ^]^ and visualized in ChimeraX 1.9.^[^ ^76^ ^]^ The remaining elements in Figure 1a were created in BioRender. Zhang, R. (2025) https://BioRender.com/oj6ftk5. b) Comparison of telomere imaging with BRIGHT, SunTag, and PP7‐PCP systems in HEK293T. Dotted lines in the top panels mark regions analyzed by Fiji to produce the fluorescence intensity plots below. Scale bar, 5 µm. c) SNR of telomere imaging with the three systems. Data are presented as means ± SD for BRIGHT (67.26 ± 29.36, *n* = 45 puncta), SunTag (10.74 ± 5.45, *n* = 45 puncta), and PP7‐PCP (9.94 ± 3.14, *n* = 45 puncta). Statistical analysis of BRIGHT SNRs with those of SunTag and PP7‐PCP was performed using the Mann‐Whitney test. *p* < 0.0001. d) SNR enhancement of BRIGHT compared to SunTag and PP7‐PCP systems. Means ± SD of SNR enhancement, BRIGHT vs SunTag = 6.26 ± 2.73; BRIGHT vs PP7‐PCP = 6.76 ± 2.95. Box plots show the median, and the whiskers extend to the minimum and maximum values. e) Histogram of telomere counts per single HEK293T cell obtained using BRIGHT (*n* = 21 cells). f) Box plot showing the number of telomere puncta detected per cell using BRIGHT. Data are presented as mean ± SD (49.48 ± 20.61 puncta per cell, *n* = 21 cells).

Here, we propose a live‐cell imaging system, bivalent near‐infrared nanobody‐mediated cascade illumination of genomic loci for high‐SNR tracking (BRIGHT), allowing robust visualization of non‐repetitive sequences. This strategy builds on recently developed bispecific and antigen‐dependent near‐infrared fluorescent nanobodies (NIR‐Fb), which are highly unstable and self‐degraded by the ubiquitin‐proteasome system unless both antigen‐binding arms are saturated.^[^ ^24^ ^]^ Based on this design, we engineered a bivalent NIR‐Fb targeting two identical 13‐amino‐acid ALFA tags (Bi‐NIR‐Fb_ALFA_).^[^ ^25^ ^]^ When dCas9 is fused with a tandem ALFA tag (dCas9‐n×ALFA), it can recruit multiple Bi‐NIR‐Fb_ALFA_ to DNA targets, whose bivalent binding further crosslinks with additional dCas9‐n×ALFA, forming multilayered assemblies that drive cascade amplification of the NIR signal. In addition, the antigen‐dependent illumination property ensures that only Bi‐NIR‐Fb_ALFA_ saturated with two ALFA tags remains fluorescent, effectively suppressing background and specifically confining fluorescence to the target locus. Overall, the cascade amplification and background denoising together contribute to the high‐SNR imaging of non‐repetitive sequences in the BRIGHT system (**Figure** **1**a).

## 2 Results

### 2.1 Design of BRIGHT Imaging System

To image non‐repetitive sequences with high SNR, we developed the BRIGHT system involving dCas9‐n×ALFA (*n* = 1, 4, 8, 16, 24) fusion proteins (Figure S1, Supporting Information), sgRNA, and Bi‐NIR‐Fb_ALFA_ (Figure 1a). Bi‐NIR‐Fb_ALFA_ plays a central role in high SNR through two key functions: 1) acting as “two hands” to gather two copies of dCas9‐n×ALFA; 2) acting as “light bulbs” remaining dark until docked into specific “sockets”—the ALFA tags. The imaging mechanism of the BRIGHT system relies on cascade amplification and signal denoising. Under the guidance of sgRNA, dCas9‐n×ALFA specifically and stably binds its DNA targets to establish an anchoring layer and recruit Bi‐NIR‐Fb_ALFA_.^[^ ^26^ ^]^ With two antigen‐binding arms, Bi‐NIR‐Fb_ALFA_ can recruit additional free dCas9‐n×ALFA toward the anchoring layer through high‐affinity antigen‐antibody interactions. The cascade assembly between Bi‐NIR‐Fb_ALFA_ and dCas9‐n×ALFA at target sites drives local NIR accumulation, resulting in strong signal amplification. Simultaneously, ALFA‐unsaturated Bi‐NIR‐Fb_ALFA_ is rapidly degraded through its antigen‐dependent stabilization, enabling minimal background (Figure 1a).

### 2.2 Functional Verification of Bi‐NIR‐Fb_ALFA_ for Imaging

As the key to high‐SNR imaging, we first confirmed Bi‐NIR‐Fb_ALFA_’s two essential properties. To assess antigen‐dependent illumination, we transfected CMV‐driven Bi‐NIR‐Fb_ALFA_ with either dCas9‐24×ALFA or dCas9‐24×GCN4 into HEK293T cells (Figure S2, Supporting Information). Strong NIR fluorescence was observed only with ALFA tags, which protect Bi‐NIR‐Fb_ALFA_ from proteasomal degradation, whereas the unrelated GCN4 tags provided no such stabilization (Figure S2d, Supporting Information). In addition, we observed nuclear NIR condensates that could be misinterpreted as specific signals (Figure S2d, Supporting Information), likely due to CMV‐driven overexpression causing crosslink with dCas9‐24×ALFA independently of DNA targets. Therefore, we optimized the promoter for appropriate Bi‐NIR‐Fb_ALFA_ expression. We tested three promoters, including EF‐1α (moderate), hPGK (moderate), and miniCMV (weak) (Figure S3a, Supporting Information). The mRNA and NIR fluorescence levels consistently indicated the highest expression from the CMV promoter, followed by EF‐1α, hPGK, and miniCMV (Figure S3b–e, Supporting Information). The hPGK promoter was identified as optimal, producing relatively low fluorescence without condensate formation or nucleolar enrichment (Figure S3d, Supporting Information).

To validate the cascade amplification mediated by bivalent binding, we constructed a monovalent version of antigen‐dependent NIR‐Fb_ALFA_ (Mo‐NIR‐Fb_ALFA_) carrying two miRFP670nano3 (Figure S4a,b, Supporting Information) and compared its labeling performance at telomeres (a classical transcriptionally silent heterochromatic locus) with Bi‐NIR‐Fb_ALFA_ co‐expressed with dCas9‐24×ALFA (Figure S4c, Supporting Information). Due to its monovalent nature, Mo‐NIR‐Fb_ALFA_ enables linear amplification, whereas Bi‐NIR‐Fb_ALFA_ forms cascade assemblies that enhance NIR fluorescence. As expected, telomere labeling with Bi‐NIR‐Fb_ALFA_ produced distinct and brighter NIR puncta, showing a ≈2.51‐fold enhancement of mean fluorescence intensity than Mo‐NIR‐Fb_ALFA_, which can be attributed to its bivalently binding capability since both constructs carry two miRFP670nano3 molecules (Figure S4d–f, Supporting Information).

### 2.3 BRIGHT Enables High‐SNR Imaging of Repetitive Genomic Loci

To explore the imaging performance of BRIGHT, we compared BRIGHT‐mediated telomere labeling (dCas9‐24×ALFA/sgTelomere/Bi‐NIR‐Fb_ALFA_) with linear amplification‐based imaging systems in HEK293T cells, including SunTag (dCas9‐24×GCN4/sgTelomere/scFv‐sfGFP)^[^ ^12^, ^13^ ^]^ and PP7‐PCP (dCas9/2×PP7‐sgTelomere/PCP‐3×GFP)^[^ ^10^ ^]^ systems (Figure 1b). BRIGHT generated telomere‐like NIR signals uniformly across the nucleus with minimal background, whereas negative groups expressing only two BRIGHT components and Bi‐NIR‐Fb_ALFA_ together with dCas9‐24×ALFA and sgGAL4 (lacking targets in human cells), displayed no signals (Figure S5, Supporting Information). These results indicate that BRIGHT labeling requires all three components and occurs preferentially at dCas9‐targeted DNA sites. We elaborate on the underlying mechanism of such selectivity as follows: once guided by sgRNA, dCas9‐n×ALFA/sgRNA stably binds to target DNA,^[^ ^27^ ^]^ providing a stable nucleation site for Bi‐NIR‐Fb_ALFA_‐mediated cascade crosslink. In contrast, dCas9‐n×ALFA/sgRNA complexes dwell for less than 1s on non‐target DNA,^[^ ^28^ ^]^ while free dCas9‐n×ALFA and Bi‐NIR‐Fb_ALFA_ remain highly dynamic and freely diffusive at low concentrations and interact through random diffusion and collisions,^[^ ^29^ ^]^ preventing stable cascade NIR condensate formation. BRIGHT achieved a maximum SNR of 154.96, with average SNR ≈6.26‐fold and ≈6.76‐fold higher than SunTag and PP7‐PCP, respectively (Figure 1b–d). To examine its labeling effectiveness, we quantified telomere counts identified by BRIGHT, which yielded an average of 49.48 ± 20.61 (mean ± standard deviation (SD)) puncta per cell (Figure 1e,f), comparable to that obtained with PP7‐PCP (49.17 ± 16.21 puncta per cell) (Figure S6, Supporting Information). Overall, BRIGHT outperformed both systems in SNR while maintaining comparable labeling efficiency.

**Figure 2:**
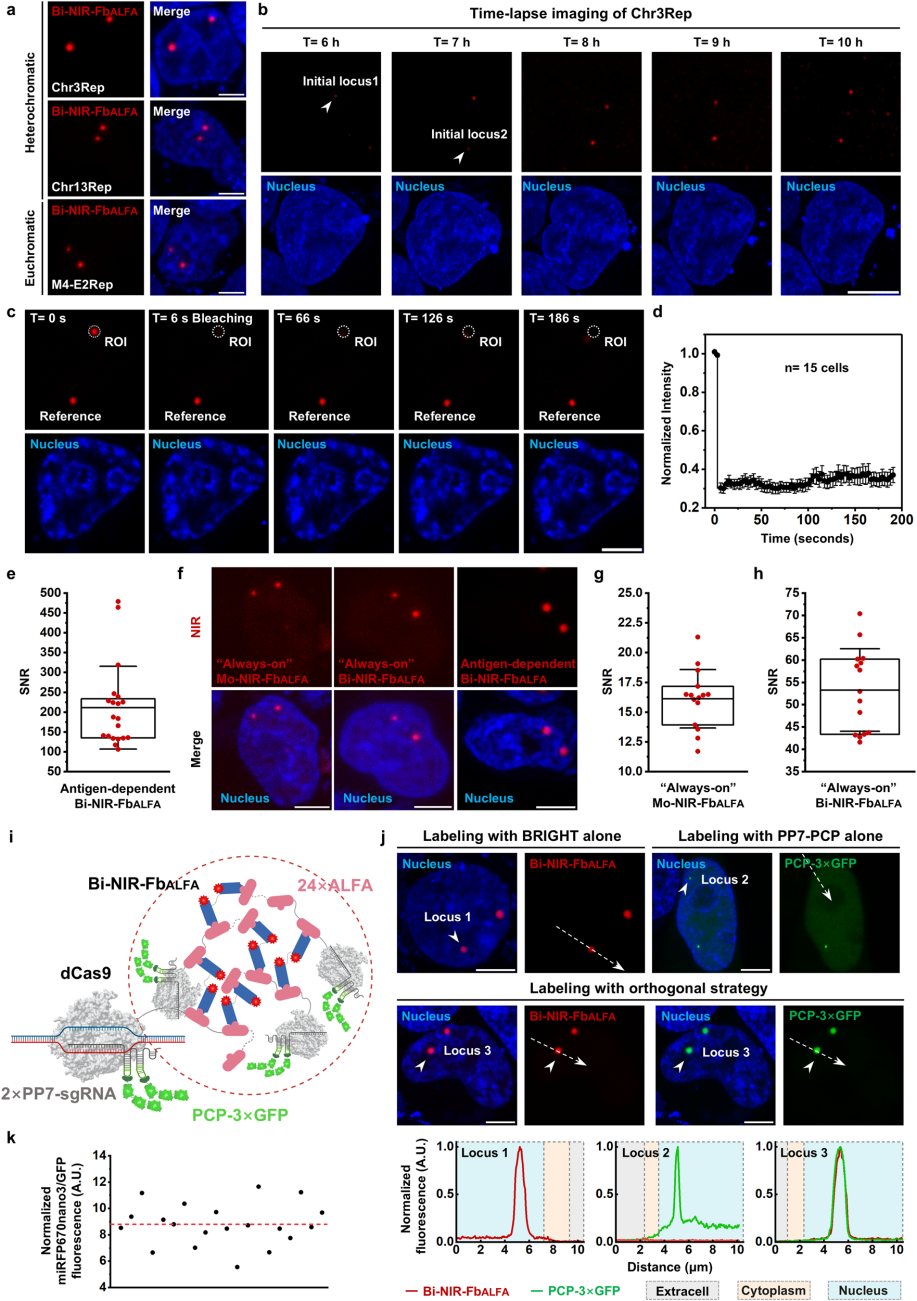
Visualization of repetitive sequences and comprehensive characterization of BRIGHT. a) Representative BRIGHT images of various high‐copy genomic loci within the heterochromatic or euchromatic regions. Scale bar, 5 µm. b) Time‐lapse images showing BRIGHT puncta formation at Chr3Rep. Scale bar, 5 µm. c,d) FRAP analysis of BRIGHT dots. Representative images (c) and fluorescence recovery curve (d) of the normalized fluorescence intensity of BRIGHT dots before and after photobleaching. *n* = 15 individual FRAP events. Error bars, means ± SEM. Scale bar, 5 µm. e) SNR analysis of Chr3Rep labeled with dCas9‐24×ALFA and antigen‐dependent Bi‐NIR‐Fb_ALFA_ in HEK293T cells. Data are presented as means ± SD (211.23 ± 104.46 with the maximum of 479.03, *n* = 20 puncta). f) Representative images of Chr3Rep labeled with dCas9‐24×ALFA and “always‐on” Mo‐NIR‐Fb_ALFA_, “always‐on” Bi‐NIR‐Fb_ALFA_ and antigen‐dependent Bi‐NIR‐Fb_ALFA_. Scale bar, 5 µm. g) SNR analysis of Chr3Rep labeled with dCas9‐24×ALFA and “always‐on” Mo‐NIR‐Fb_ALFA_ in HEK293T cells. Data are presented as means ± SD (16.13 ± 2.46, *n* = 15 puncta). h) SNR analysis of Chr3Rep labeled with dCas9‐24×ALFA and “always‐on” Bi‐NIR‐Fb_ALFA_ in HEK293T cells. Data are presented as means ± SD (53.29 ± 9.26, *n* = 15 puncta). i) Schematic diagram of orthogonal strategy. Protein structures were predicted with AlphaFold3^[^ ^75^ ^]^ and visualized in ChimeraX 1.9.^[^ ^76^ ^]^ The remaining elements in Figure 2i were created in BioRender. Zhang, R. (2025) https://BioRender.com/wqskud0. j) Representative images of Chr3Rep labeled with BRIGHT alone, PP7‐PCP alone, and the orthogonal strategy in HEK293T cells. Dotted arrows in the top panels mark regions analyzed by Fiji to produce the fluorescence intensity plots below. Scale bar, 5 µm. k) Quantifications of the dual‐normalized miRFP670nano3/GFP fluorescence ratio on dCas9‐24×ALFA. Ratios were first normalized to the correction factor calculated from the GFP‐miRFP670nano3 fusion protein to correct for intrinsic intensity differences between the two FPs^[^ ^12^ ^]^ (Figure S11, Supporting Information). And further normalized according to the molecular stoichiometry, each dCas9‐24×ALFA/2×PP7‐sgRNA complex recruits six GFP molecules via two PCP‐3×GFP, while each Bi‐NIR‐Fb_ALFA_ carries two miRFP670nano3, corresponding to a normalization factor of three. *n* = 20 puncta. Each dot represents a single punctum, and the red dashed line indicates the average value. Identical imaging conditions with Figure S11 (Supporting Information) were used for the calculations of the normalized Bi‐NIR‐Fb_ALFA_ occupancy.

We next asked whether BRIGHT could label diverse repetitive genomic loci beyond telomeres. HEK293T cells were transfected with dCas9‐24×ALFA, Bi‐NIR‐Fb_ALFA_, and sgRNAs targeting high‐copy loci (>100 copies) on Chromosome 3 (Chr3Rep), Chromosome 13 (Chr13Rep), and exon 2 of the *MUC4* gene (M4‐E2Rep) (**Figure** **2**a; Table S3, Supporting Information). Furthermore, we tested the performance of BRIGHT on low‐copy loci (3‐100 copies) across chromosomes 7, 9, 10, 13, 17, 19, 21 and X. Based on chromatin structure and spatial positioning relative to the nuclear lamina,^[^ ^30^, ^31^, ^32^ ^]^ these loci were classified into two groups, 1) lamina‐associated domains (LADs) located in transcriptionally silent heterochromatic regions, including high‐copy loci such as Chr3Rep and Chr13Rep, as well as low‐copy loci such as Chr21Rep, Chr7‐*DPP6*, Chr19Rep, Chr19‐*TDRD12* and Chr10‐*DOCK1*, according to the CHIP‐seq datasets for H3K9me3 and H3K27ac modifications in HEK293T cells from the ENCODE project (GEO, GSE174869; GEO:GSE174866); 2) non‐LADs located in transcriptionally active euchromatic regions, including Chr17‐*LINC20887*, Chr9‐*PNPLA7*, Chr13‐*POLR1D* and ChrX‐*XIST* (ENCODE project, GEO, GSE174869; GEO:GSE174866). All tested loci were successfully labeled, exhibiting distinct NIR puncta consistent with their copy numbers in HEK293T cells (Figure S7 and Table S3, Supporting Information).

To further characterize the intrinsic properties of BRIGHT, Chr3Rep was selected as a representative target. We conducted time‐lapse imaging to monitor the temporal dynamics of BRIGHT. Imaging analysis revealed that NIR foci appeared as early as 6 h post‐transfection and gradually increased in size and intensity over time in HEK293T cells (Figure 2b). Notably, during continuous tracking for up to 72 h, these foci remained almost unchanged, demonstrating the high temporal stability of BRIGHT (Figure S8, Supporting Information). Long‐term and continuous tracking raises concerns about potential phototoxic effects on cellular function. To assess this, we monitored reactive oxygen species (ROS) production, an early and sensitive marker of phototoxicity.^[^ ^33^ ^]^ During continuous excitation for 15 cycles of single optical sections at 5s intervals, BRIGHT‐labeled cells exhibited only a slight ROS increase compared to the negative control, indicating low phototoxicity (Figure S9, Supporting Information). This likely benefits from the use of the NIR fluorescent protein miRFP670nano3, consistent with reports that NIR excitation minimizes phototoxicity in living cells.^[^ ^34^ ^]^

We subsequently conducted fluorescence recovery after photobleaching (FRAP) experiments to determine the binding dynamics of BRIGHT components.^[^ ^35^ ^]^ In FRAP experiments, the regions of interest (ROIs) were bleached for 0.12–0.24 s using a 640‐nm laser at 33% power, and fluorescence recovery was monitored every 3 s for 180 s post‐bleaching.^[^ ^18^, ^36^ ^]^ Under these conditions, bleached BRIGHT puncta showed negligible recovery (Figure 2c,d), indicating minimal exchange between bound and free Bi‐NIR‐Fb_ALFA_,^[^ ^35^ ^]^ likely resulting from the stable binding of Bi‐NIR‐Fb_ALFA_ within the cascade amplification complex and substantial degradation of unbound Bi‐NIR‐Fb_ALFA_. Such stable binding may explain why BRIGHT puncta can still be observed during continuous tracking for up to 72 h (Figure S8, Supporting Information), which may have important implications for studies investigating cell cycle‐dependent or differentiation‐associated chromatin dynamics, and tracking viral intra‐nuclear events at the single‐cell level.^[^ ^31^, ^37^, ^38^, ^39^, ^40^ ^]^ It's noteworthy that minimal exchange between bound and free Bi‐NIR‐Fb_ALFA_ may restrict high‐frequency tracking, as photobleaching at a labeled locus cannot be effectively replenished by diffusing molecules.^[^ ^41^ ^]^ Thus, decreased exposure intensity and duration may help to mitigate this effect.

We next evaluated SNR in BRIGHT labeling by targeting Chr3Rep in HEK293T cells using dCas9‐24×ALFA and Bi‐NIR‐Fb_ALFA_. Leveraging the bivalent binding and antigen‐dependent illumination of Bi‐NIR‐Fb_ALFA_, BRIGHT achieved a high SNR of 211.23 ± 104.46 (mean ± SD) (Figure 2e,f). To dissect the contributions of each function, we generated an “always‐on” Mo‐NIR‐Fb_ALFA_ lacking both functions, and an “always‐on” Bi‐NIR‐Fb_ALFA_ retaining bivalent binding but lacking antigen‐dependent illumination (Figure S10a–c, Supporting Information). We then labeled Chr3Rep in HEK293T cells with dCas9‐24×ALFA and the two “always‐on” nanobodies and yielded SNRs of 16.13 ± 2.46 and 53.29 ± 9.26, corresponding to a ≈3.38‐fold enhancement from bivalent binding (Figure 2f–h; Figure S10d, Supporting Information). The contribution of antigen‐dependent illumination was assessed by comparing SNRs between “always‐on” (53.29 ± 9.26) and antigen‐dependent Bi‐NIR‐Fb_ALFA_ (211.23 ± 104.46), resulting in a ≈4.08‐fold increase (Figure 2e,h; Figure S10e, Supporting Information). These results indicate that both bivalent binding and antigen‐dependent illumination play important roles in SNR enhancement.

To quantify Bi‐NIR‐Fb_ALFA_ occupancy on dCas9‐24×ALFA within the cascade complex, we designed an orthogonal BRIGHT/PP7‐PCP strategy that forms dCas9‐24×ALFA/Bi‐NIR‐Fb_ALFA_/2×PP7‐sgChr3Rep/PCP‐3×GFP complexes and enables Bi‐NIR‐Fb_ALFA_ occupancy estimation from the miRFP670nano3/GFP fluorescence intensity ratios (Figure 2i–j). Ratios were dual‐normalized to correct intrinsic FP intensity differences^[^ ^12^ ^]^ and molecular stoichiometry (Figure 2k; Figure S11, Supporting Information). The average occupancy number was 8.78 ± 1.60 Bi‐NIR‐Fb_ALFA_ per dCas9‐24×ALFA (36.58% (8.78/24) occupancy rate; Figure 2k). Given that PCP‐3×GFP binding is not 100% efficient, the actual occupancy is higher.

### 2.4 BRIGHT Allows High‐SNR Visualization of Endogenous and Exogenous Non‐Repetitive Sequences

**Figure 3:**
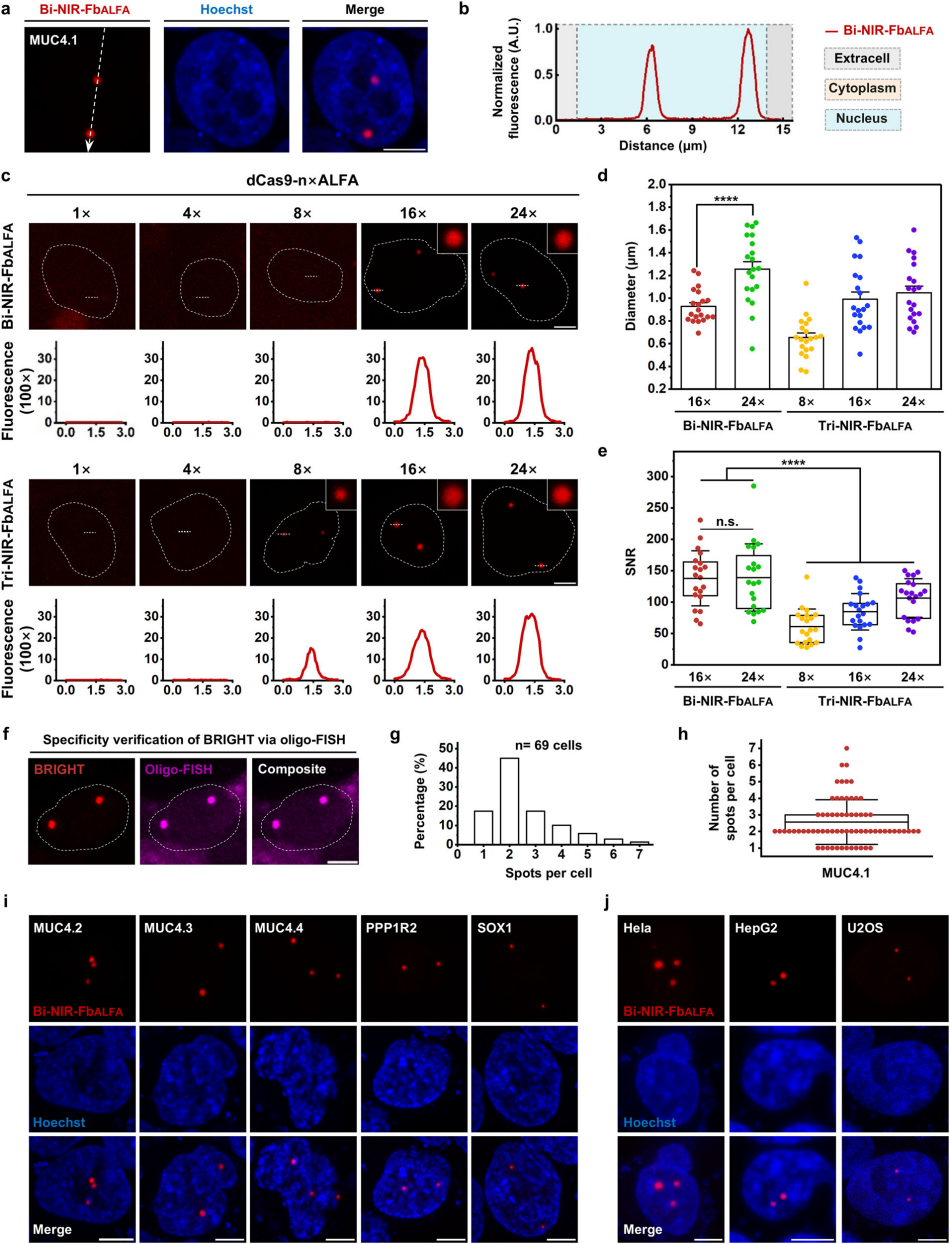
Visualization of endogenous non‐repetitive loci using BRIGHT. a,b) Representative images of MUC4.1 labeling with BRIGHT alone in HEK293T cells. Dotted arrow in (a) marks regions analyzed by Fiji to produce the fluorescence intensity plot in (b). Scale bar, 5 µm. c) Representative images of MUC4.1 labeling performed with dCas9 fused to varying numbers of ALFA repeats (1× to 24×), combined with either Bi‐NIR‐Fb_ALFA_ or Tri‐NIR‐Fb_ALFA_. Image acquisition was performed under identical conditions for fluorescence intensity and SNR analysis. The white dotted circle line represents the nuclear. Dotted lines in the top panels mark regions analyzed by Fiji to produce the fluorescence intensity plots below. Scale bar, 5 µm. d) Diameter analysis of MUC4.1 dots labeled with dCas9‐n×ALFA in combination with either Bi‐NIR‐Fb_ALFA_ or Tri‐NIR‐Fb_ALFA_. Data are presented as means ± SEM (0.93 ± 0.03 µm and 1.26 ± 0.07 µm for Bi‐NIR‐Fb_ALFA_/dCas9‐16×ALFA and Bi‐NIR‐Fb_ALFA_/dCas9‐24×ALFA, respectively; 0.65 ± 0.04 µm, 0.99 ± 0.06 µm and 1.05 ± 0.06 µm for Tri‐NIR‐Fb_ALFA_/dCas9‐8×ALFA, Tri‐NIR‐Fb_ALFA_/dCas9‐16×ALFA and Tri‐NIR‐Fb_ALFA_/dCas9‐24×ALFA, respectively). *n* = 20 puncta for each group. Statistical analysis of diameters between Bi‐NIR‐Fb_ALFA_/dCas9‐16×ALFA and Bi‐NIR‐Fb_ALFA_/dCas9‐24×ALFA was performed using the Mann‐Whitney test. *p* < 0.0001. e) SNR analysis of MUC4.1 dots in the above groups. Data are presented as means ± SD (137.87 ± 43.89 and 139.19 ± 53.67 for Bi‐NIR‐Fb_ALFA_/dCas9‐16×ALFA and Bi‐NIR‐Fb_ALFA_/dCas9‐24×ALFA, with maximum values of 230.57 and 284.84, respectively; 60.99 ± 28.00, 84.58 ± 28.94 and 106.56 ± 30.85 for Tri‐NIR‐Fb_ALFA_/dCas9‐8×ALFA, Tri‐NIR‐Fb_ALFA_/dCas9‐16×ALFA and Tri‐NIR‐Fb_ALFA_/dCas9‐24×ALFA, with the maximum values of 139.81, 138.62 and 150.27, respectively). *n* = 20 puncta for each group. Statistical analysis of SNRs between Bi‐NIR‐Fb_ALFA_ and Tri‐NIR‐Fb_ALFA_ labeling was performed using the Mann‐Whitney test (*p* < 0.0001), and comparisons between Bi‐NIR‐Fb_ALFA_/dCas9‐16×ALFA and Bi‐NIR‐Fb_ALFA_/dCas9‐24×ALFA were performed using the two‐tailed Student's *t*‐test (*p* > 0.5). f) Representative images showing BRIGHT‐labeled MUC4.1 loci co‐localized with oligo‐FISH signals to validate labeling specificity. The white dotted circle line represents the nuclear. Scale bar, 5 µm. g) Histogram of MUC4.1 counts per single HEK293T cell obtained using BRIGHT. *n* = 69 cells. h) Box plot showing the number of MUC4.1 puncta detected per cell using BRIGHT. Data are presented as mean ± SD (2.57 ± 1.34 puncta per cell, *n* = 69 cells). i) Representative BRIGHT images of various non‐repetitive loci on Chromosomes 3 and 13. Scale bar, 5 µm. j) Representative BRIGHT images of MUC4.1 loci in living human cell lines of diverse tissue origins and disease types. Scale bar, 5 µm.

To realize the ultimate goal of visualizing non‐repetitive sequences, we applied dCas9‐24×ALFA and Bi‐NIR‐Fb_ALFA_ to label the non‐repetitive MUC4.1 locus within the *MUC4* gene (Table S3, Supporting Information). HEK293T cells transfected with BRIGHT components showed distinct NIR MUC4.1 signals (**Figure** **3**a,b), whereas no puncta were observed with sgGAL4 (Figure S12, Supporting Information), demonstrating successful visualization of a non‐repetitive sequence.

We then optimized conditions for non‐repetitive sequences visualization. First, we evaluated the labeling performance of dCas9 fused with varying numbers of ALFA tags (dCas9‐1×, 4×, 8×, 16×, or 24×ALFA) using Bi‐NIR‐Fb_ALFA_. Detectable MUC4.1 signals appeared only with dCas9‐16×ALFA and dCas9‐24×ALFA (Figure 3c). MUC4.1 labeled with dCas9‐16×ALFA produced significantly smaller puncta (*p* < 0.0001; Figure 3d), while both constructs showed similarly high SNR (137.87 ± 43.89 vs 139.19 ± 53.67, mean ± SD; *p* > 0.05; Figure 3e). We then quantified Bi‐NIR‐Fb_ALFA_ occupancy on dCas9‐16×ALFA using the orthogonal strategy (Figure 2i), yielding the binding of at least 7.85 ± 1.29 Bi‐NIR‐Fb_ALFA_ (49.1% occupancy; Figure S13, Supporting Information). This was comparable to the Bi‐NIR‐Fb_ALFA_ occupancy on dCas9‐24×ALFA (Figure 2k), indicating that increasing ALFA repeats beyond 16 does not enhance Bi‐NIR‐Fb_ALFA_ recruitment, likely due to steric hindrance saturation, which can account for the similar SNR of MUC4.1 labeling with dCas9‐16×ALFA and dCas9‐24×ALFA. Next, we tested whether increasing the valency of NIR‐Fb_ALFA_ improved labeling performance by constructing a trivalent version of NIR‐Fb_ALFA_ (Tri‐NIR‐Fb_ALFA_). Although Tri‐NIR‐Fb_ALFA_ labeled MUC4.1 with dCas9 fused to ≥8×ALFA (Figure 3c), its overall SNR was significantly lower than Bi‐NIR‐Fb_ALFA_ (*p* < 0.0001; Figure 3e), likely due to its requirement for simultaneous saturation of three binding arms, where incomplete binding may cause local degradation and reduced signal fluorescence (Figure S14, Supporting Information). Thus, dCas9‐16×ALFA with Bi‐NIR‐Fb_ALFA_ is the preferred choice for labeling non‐repetitive sequences. Similarly, we then assessed the contributions of the bivalent binding and antigen‐dependent illumination of Bi‐NIR‐Fb_ALFA_ in non‐repetitive MUC4.1 labeling using dCas9‐16×ALFA. “Always‐on” Mo‐NIR‐Fb_ALFA_ produced no detectable signal, whereas “always‐on” Bi‐NIR‐Fb_ALFA_ enabled imaging with an SNR of 44.61 ± 13.27 (Figure S15a,b, Supporting Information). Antigen‐dependent Bi‐NIR‐Fb_ALFA_ achieved a ≈3.42‐fold SNR enhancement over “always‐on” Bi‐NIR‐Fb_ALFA_ (Figure S15c, Supporting Information). These results indicate that bivalent binding‐mediated cascade amplification is essential for non‐repetitive sequence imaging, with antigen‐dependent illumination providing additional SNR enhancement.

We subsequently investigated the labeling specificity and efficiency of MUC4.1 with Bi‐NIR‐Fb_ALFA_/dCas9‐16×ALFA. After live‐cell imaging of MUC4.1, HEK293T cells were fixed for oligo‐FISH targeting M4‐E2Rep repetitive regions. BRIGHT‐labeled MUC4.1 puncta showed clear co‐localization with FISH signals (Figure 3f), confirming the high specificity of BRIGHT. Furthermore, we frequently observed 2–3 labeled MUC4.1 puncta per cell (2.57 ± 1.34 puncta per cell, mean ± SD). This is consistent with the reported 2–3 copies of *MUC4* gene in HEK293T cells,^[^ ^19^, ^42^ ^]^ supporting the high BRIGHT labeling efficiency (Figure 3g,h).

We further asked whether BRIGHT is versatile for labeling other non‐repetitive sequences and applicable across cell lines. Five additional non‐repeating loci in HEK293T cells were tested, including MUC4.2, MUC4.3, and MUC4.4 within the *MUC4* gene, *PPP1R2* on Chromosome 3, and *SOX1* on Chromosome 13 (Figure 3i; Table S3, Supporting Information). All loci were effectively labeled, showing bright NIR puncta with minimal background. We then tested the ability of BRIGHT to label MUC4.1 in HeLa, HepG2 and U2OS cell lines. Puncta numbers were consistent with the *MUC4* gene copy number across cell types (Figure 3j). In highly aneuploid U2OS cells, 1–4 MUC4.1 puncta were observed, matching with previous report^[^ ^43^ ^]^ (Figure S16, Supporting Information). Thus, BRIGHT demonstrates broad applicability and versatility.

**Figure 4:**
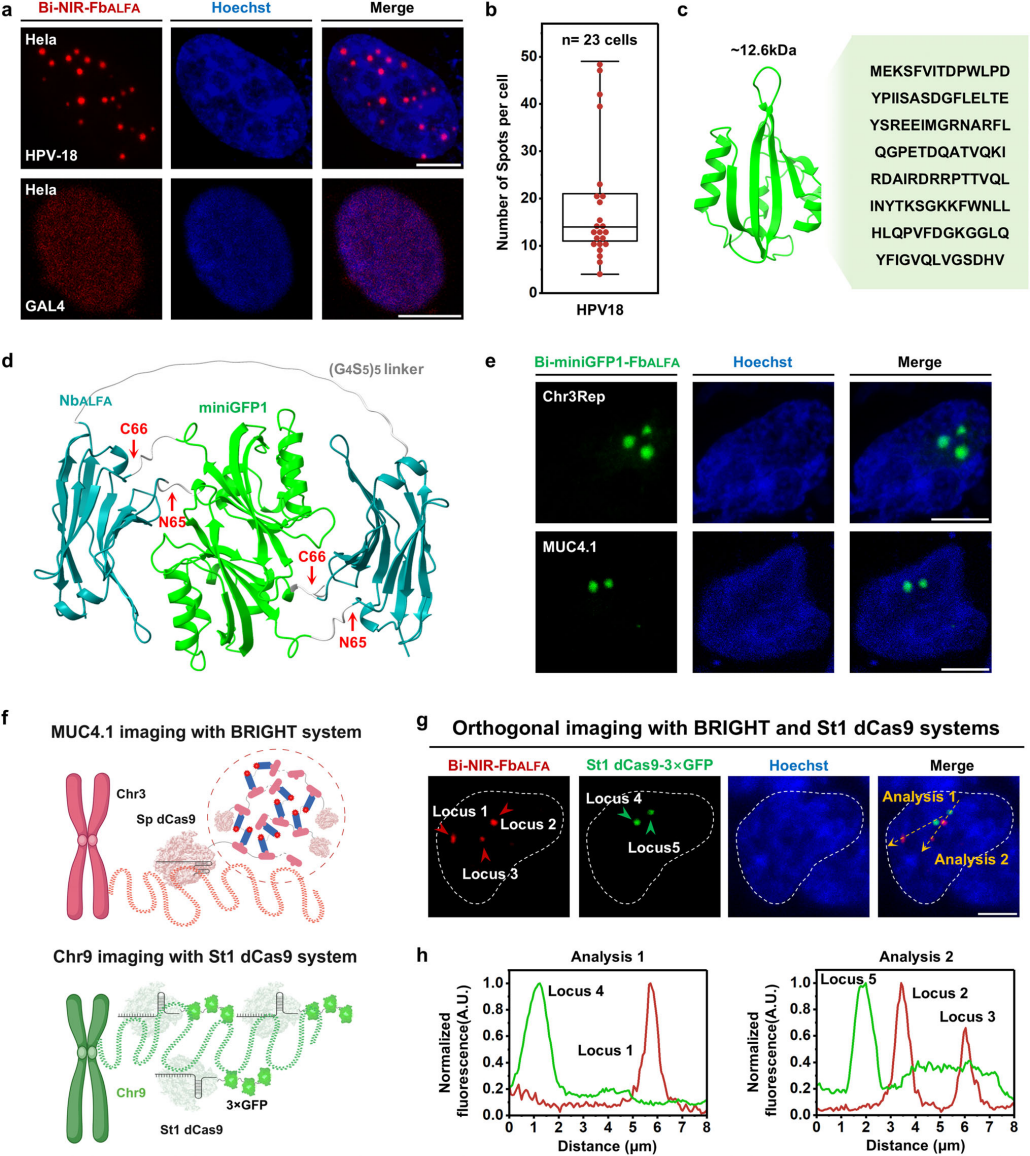
Visualization of exogenous HPV‐18 as well as multi‐color and orthogonal imaging of genomic loci. a) Representative BRIGHT images of HPV‐18 in HeLa. Scale bar, 5um. b) Box plot showing the number of HPV‐18 puncta detected per cell using BRIGHT (*n* = 23 cells). A box plot shows the median, and the whiskers extend to the minimum and maximum values. c) The amino acid sequence and structural features of miniGFP1. Protein structures were predicted with AlphaFold3^[^ ^75^ ^]^ and visualized in ChimeraX 1.9.^[^ ^76^ ^]^ d) Protein structure of Bi‐miniGFP1‐Fb_ALFA_ showing miniGFP1 insertion at the N65/C66 site. Protein structures were predicted with AlphaFold3^[^ ^75^ ^]^ and visualized in ChimeraX 1.9.^[^ ^76^ ^]^ e) Representative BRIGHT images of Chr3Rep and MUC4.1 labeling with Bi‐miniGFP1‐Fb_ALFA_/dCas9‐16×ALFA. Scale bar, 5um. f) Schematic of orthogonal imaging using BRIGHT and St1 dCas9‐3×GFP/St1 sgRNA. Protein structures were predicted with AlphaFold3^[^ ^75^ ^]^ and visualized in ChimeraX 1.9.^[^ ^76^ ^]^ The remaining elements in Figure 4f were created in BioRender. Zhang, R. (2025) https://BioRender.com/gfgay30. g,h) Representative images of orthogonal imaging of MUC4.1 and the GGAAT tandem repeat in the pericentromeric region of Chromosome 9. Red and green arrows in (g) indicate BRIGHT and St1 dCas9 dots, respectively. Dotted yellow arrows in (g) mark regions analyzed by Fiji to produce the fluorescence intensity plots in (h). Scale bar, 5um.

In addition, we validated BRIGHT's capability to detect exogenous non‐repetitive sequences by targeting the integrated high‐risk human papillomavirus 18 (HPV‐18) in the HeLa genome, a major cause of cervical cancer.^[^ ^44^ ^]^ We designed an sgRNA targeting the highly conserved L1 region, which is frequently tested in commercial polymerase chain reaction (PCR) assays,^[^ ^45^ ^]^ and confirmed its presence in the HeLa genome (Figure S17, Supporting Information). BRIGHT transfection in HeLa cells yielded 4–49 foci per cell, consistent with previously reported HPV‐18 copy numbers (**Figure** **4**a,b).^[^ ^44^, ^46^ ^]^ In contrast, no foci were detected using the sgGAL4 control, confirming labeling specificity (Figure 4a).

### 2.5 BRIGHT Could Offer Diverse Emission Spectrum for Multi‐Color and Orthogonal Genomic Loci Imaging

Multi‐color orthogonal imaging enables simultaneous visualization of multiple genomic loci within the same cell, facilitating investigations into their spatial organization, dynamic behavior, and functional interactions.^[^ ^9^, ^15^ ^]^ We thus explored whether BRIGHT could be extended beyond NIR and combined orthogonally with other imaging methods for multiplexed detection.

Since the emission spectrum of BRIGHT depends on the FP inserted at the N65/C66 split site of Bi‐Fb_ALFA_, we tested alternative FPs to broaden its spectral range while retaining its antigen‐dependent illumination and bivalent binding. Candidate FP should meet four criteria, 1) stable expression in mammalian cells, ensuring sustained brightness over time and a robust fluorescent signal;^[^ ^47^ ^]^ 2) no requirement for exogenous cofactors, avoiding incompatibility with live cells and minimizing interference with cellular physiology;^[^ ^48^, ^49^ ^]^ 3) a strictly monomeric form, preventing the formation of dimers and aggregates which could disrupt proper folding of Bi‐Fb_ALFA_;^[^ ^47^, ^50^ ^]^ 4) small size (< 20 kDa) and a compact structure with closely positioned N‐ and C‐termini, avoiding excessive structural distortion of Bi‐Fb_ALFA_ and maintaining its antigen‐binding capability.^[^ ^24^, ^51^ ^]^ The monomeric 12.6 kDa cyan fluorescent protein miniGFP1^[^ ^52^ ^]^ was selected via FPbase^[^ ^53^ ^]^ (Figure 4c) and inserted at the N65/C66 site, generating Bi‐miniGFP1‐Fb_ALFA_ (Figure4d). We then assessed whether its antigen‐dependent illumination and bivalent binding properties were preserved. Strong green fluorescence appeared only with ALFA tags, similar to Bi‐NIR‐Fb_ALFA_ (Figure S18, Supporting Information). And bright green loci were observed when labeling Chr3Rep and MUC4.1 in HEK293T cells (Figure 4e). Thus, BRIGHT fluorescence can be tuned across different wavelengths by changing FP while retaining its core functions.

To image multiple genomic loci simultaneously, we combined BRIGHT with another CRISPR‐based system. Specifically, we used Bi‐NIR‐Fb_ALFA_/dCas9‐16×ALFA to label MUC4.1 and *Streptococcus thermophilus* dCas9 fused with 3×GFP (St1 dCas9‐3×GFP) along with St1 sgRNA to target a GGAAT tandem repeat enriched in the pericentromeric region of Chromosome 9^[^ ^9^ ^]^ in HEK293T cells (Figure 4f). We observed independently distributed NIR and green fluorescent puncta (Figure 4g,h), demonstrating the feasibility of using orthogonal dCas9 variants to simultaneously visualize distinct genomic regions without cross‐talk between imaging systems.

### 2.6 BRIGHT Allows Visualization of Genomic Loci Dynamics

**Figure 5:**
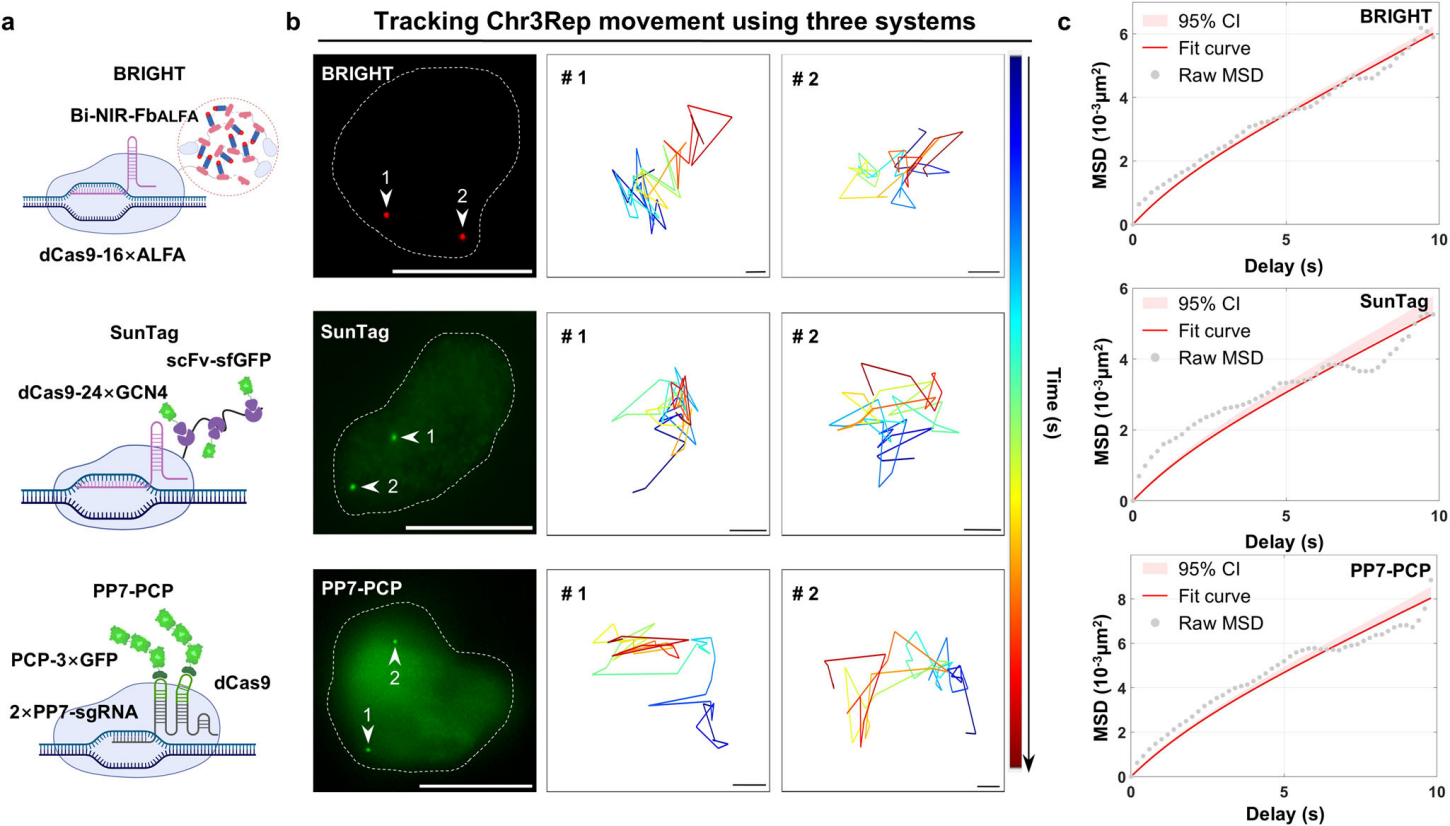
Comparison of chromatin dynamics revealed by BRIGHT and other imaging systems. a) Schematic diagrams of Chr3Rep labeling with the BRIGHT, SunTag, and PP7‐PCP systems. Elements in Figure 5a were created in BioRender. Zhang, R. (2025) https://BioRender.com/jgdsrde. b) Representative Chr3Rep images labeled using the BRIGHT, SunTag, and PP7‐PCP systems. The white dotted circle lines on the left panel represent the nuclear. White arrows annotated with locus numbers in the left panel indicate puncta chosen for subsequent single‐particle tracking, with corresponding trajectories displayed in right panels. Scale bar for fluorescence figure, 5 µm. Scale bar for the trajectories, 0.02 µm. The gradient colors of the trajectories represent time progression from 0 to 10 s. c) Average MSD curves of Chr3Rep labeled with BRIGHT, SunTag, and PP7‐PCP systems in HEK293T cells. *n* = 20 cells.

Tracking chromatin dynamics is key to revealing how the spatiotemporal regulation of genes contributes to cellular functional events.^[^ ^54^ ^]^ We asked whether BRIGHT could visualize chromatin dynamics like previously developed tools. To investigate this, we performed high‐frequency confocal imaging (0.2s per frame) of Chr3Rep motion using Bi‐NIR‐Fb_ALFA_/dCas9‐16×ALFA, and compared it with previously reported SunTag^[^ ^12^, ^13^ ^]^ and PP7‐PCP^[^ ^10^ ^]^ systems. We separately expressed BRIGHT and the two systems targeting Chr3Rep in HEK293T cells (**Figure** **5**a), and analyzed the movement by single‐particle tracking and mean‐squared displacement (MSD) analyses. We observed similar confined movements of the labeled foci with the BRIGHT, SunTag, and PP7‐PCP systems at ≈10 s timescale, with comparable effective diffusion coefficients (*D_eff_*) of 1.30 × 10^−4^, 1.13 × 10^−4^ and 1.67 ×1 0^−4^ µm2/s, respectively (Figure 5b,c; Videos S1–S3, Supporting Information). The downward fluctuations at larger time lags in raw MSD data from all three methods likely reflect statistical artifacts and biological heterogeneity, primarily due to the reduced number of trajectories available at longer lag times and trajectory heterogeneity, common sources of uncertainty in MSD analysis^[^ ^55^, ^56^, ^57^ ^]^ (Figure 5c). In a word, BRIGHT performs comparably to traditional CRISPR imaging methods in dynamic tracking, and its higher SNR enables precise signal localization.

### 2.7 BRIGHT Links Nuclear Positioning and Dynamics of Genomic Loci to Transcriptional Activity

**Figure 6:**
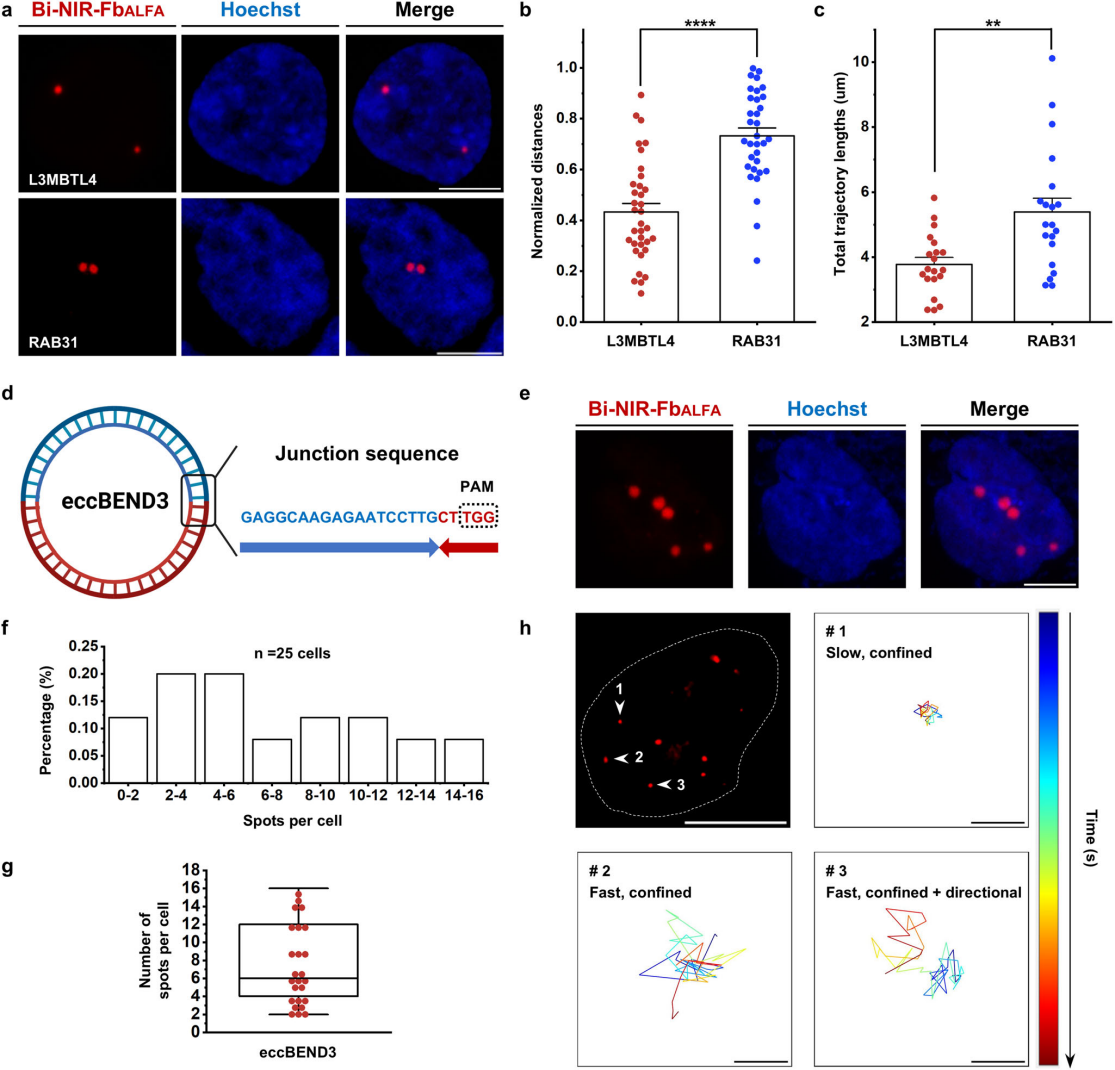
BRIGHT reveals the gene nuclear location and heterogeneity of eccDNA in living cells. a) Representative BRIGHT images of *L3MBTL4* and *RAB31* in HEK293T cells. Scale bar, 5um. b) Normalized distances of *L3MBTL4* and *RAB31* fluorescent spots. Data are presented as means ± SEM (0.43 ± 0.03 for *L3MBTL4* spots and 0.73 ± 0.03 for *RAB31* spots, respectively). Statistical analysis was performed using the two‐tailed Student's *t*‐test. *n* = 35 puncta for *L3MBTL4* group and 33 puncta for *RAB31* group. *p* < 0.0001. c) Total trajectory lengths of *L3MBTL4* and *RAB31* fluorescent signals from 0 to 10 s. Data are presented as means ± SEM (3.78 ± 0.21 µm for *L3MBTL4* spots and 5.39 ± 0.42 µm for *RAB31* spots, respectively). Statistical analysis was performed using the Mann‐Whitney test. *n* = 20 puncta for each group. *p* < 0.01. d) Schematic strategy for eccBEND3 labeling using BRIGHT. Elements in Figure 6d were created in BioRender. Zhang, R. (2025) https://BioRender.com/4nbpypg. e) Representative BRIGHT images of eccBEND3 labeling in HepG2 cells. Scale bar, 5um. f) Histogram of eccBEND3 counts per single HepG2 cell obtained using BRIGHT (*n* = 25 cells).g) Box plot showing the number of eccBEND3 puncta detected per cell using BRIGHT. A box plot shows the median, and the whiskers extend to the minimum and maximum values. *n* = 25 cells. h) Representative trajectories for eccBEND3 revealing the heterogeneity of eccDNA movements. The time interval is 0.2 s, and the gradient color of each trajectory represents the time progression from 0 to 10 s. Scale bar for fluorescence figure, 5 µm. Scale bar for the trajectories, 0.05 µm.

The nuclear positioning and dynamics of genes are associated with their transcriptional activity.^[^ ^58^ ^]^ LADs, which interact with the nuclear lamina and localize to the nuclear periphery, are typically transcriptionally silent with constrained motion. In contrast, non‐LADs are centrally positioned and transcriptionally active with greater mobility.^[^ ^30^, ^59^ ^]^ Given this association, we explored the applicability of BRIGHT for visualizing the nuclear positions of LADs and non‐LADs. Based on chromatin immunoprecipitation sequencing (ChIP‐seq) datasets for H3K9me3 and H3K36me3 modifications in HEK293T cells from the ENCODE project (GEO, GSE174869 and GSE174870), the *L3MBTL4* gene (a LAD‐associated site marked by H3K9me3) and the *RAB31* gene (a non‐LAD‐associated site marked by H3K36me3) in HEK293T cells were selected as representative targets (Figure S19a, Supporting Information)^[^ ^60^ ^]^ Reverse transcription quantitative PCR (RT‐qPCR) confirmed higher transcriptional activity of *RAB31* compared to *L3MBTL4*, consistent with ENCODE annotations (Figure S19b, Supporting Information). We then labeled these two loci with BRIGHT to visualize their nuclear positioning and track the movements. The distances from the labeled puncta to the nuclear periphery were measured and normalized to nuclear diameter to enable comparison (Figure S19c, Supporting Information). Fluorescent signals corresponding to *L3MBTL4* were significantly closer to the nuclear periphery compared to *RAB31* (*p* < 0.0001; **Figure** **6**a,b). Time‐lapse imaging was conducted to track the spatiotemporal dynamics of the two loci (Videos S4 and S5, Supporting Information) and found that *RAB31* exhibited greater total trajectory lengths and spatial ranges than *L3MBTL4* over the same time period (*p* < 0.01; Figure 6c), reflecting its higher mobility and dynamic activity. These findings demonstrate the utility of the BRIGHT system in linking nuclear location and dynamics of genomic loci with their transcriptional activity.

### 2.8 BRIGHT Detects eccDNA and Visualizes its Heterogeneity

EccDNA is a class of circular DNA molecules independent of chromosomal genomes.^[^ ^3^ ^]^ Due to the absence of centromeres, eccDNA undergoes uneven segregation during mitosis,^[^ ^61^ ^]^ leading to pronounced intercellular copy number variability and further promoting intratumoral heterogeneity.^[^ ^62^ ^]^ To directly visualize such heterogeneity in live cells, we applied BRIGHT for high‐SNR imaging of eccDNA. As previously reported, eccBEND3 is present in HepG2 cells.^[^ ^17^ ^]^ We extracted total eccDNA from HepG2 cells and verified the eccBEND3 junction sequence using breakpoint‐specific PCR and Sanger sequencing (Figure S20, Supporting Information). The junction sequence, unique and absent in parental chromosomal DNA, was selected for BRIGHT labeling (Figure 6d,e). BRIGHT imaging revealed heterogeneous eccBEND3 copy number, in agreement with earlier observations.^[^ ^17^ ^]^ Specially, unlike genome loci with relatively consistent copy numbers, eccBEND3 exhibited marked cell‐to‐cell variability, ranging from 2 to 16 copies per cell (Figure 6f,g). In addition to the high variability in copy number, we also observed dynamic heterogeneity of eccDNA (Figure 6h; Video S6, Supporting Information). Three characterized eccBEND3 signals within the same cell exhibited distinct movement lengths of 0.273 µm for spot 1, 1.068 µm for spot 2, and 0.928 µm for spot 3, with spot 2 showing the most pronounced positional translocation. Besides, spots 1 and 2 show confined diffusion, while spot 3 displays an obvious intermittent hopping. The heterogeneity in motion dynamics, together with the variability in eccDNA copy number, shapes a complex and heterogeneous eccDNA ecosystem that enhances tumor cell adaptability under therapeutic pressure.^[^ ^63^ ^]^ BRIGHT enables direct visualization of eccDNA heterogeneity, offering a novel tool to investigate eccDNA behavior and disease mechanisms.

## 3 Discussion and Conclusion

Here, we report BRIGHT, a novel live‐cell imaging strategy for high‐SNR imaging of non‐repetitive sequences. BRIGHT leverages the cascade‐dependent illumination driven by two unique biochemical properties of Bi‐NIR‐Fb_ALFA_, bivalent binding ability and antigen‐dependent illumination. High signal output is achieved through multivalent crosslinking between tandem ALFA tags and Bi‐NIR‐Fb_ALFA_, while background noise is minimized through the self‐degradation of unbound Bi‐NIR‐Fb_ALFA_ (Figure 1a). We have experimentally substantiated the BRIGHT mechanisms throughout the manuscript. First, we demonstrated that Bi‐NIR‐Fb_ALFA_ is specifically ALFA‐dependent (Figure S2, Supporting Information), and its bivalent binding promotes cascade complex formation and yields a ≈2.51‐fold higher fluorescence intensity than Mo‐NIR‐Fb_ALFA_ under identical fluorophore stoichiometry (Figure S4, Supporting Information). We then dissected the contributions of the two functions to SNR enhancement at Chr3Rep, showing that both play important roles (3.38 ± 0.54‐flod enhancement for bivalent binding and 4.08 ± 0.70‐fold enhancement for background suppression; Figure 2e–h; Figure S10d,e, Supporting Information). For non‐repetitive loci such as MUC4.1, cascade amplification was essential and background suppression further improved the SNR (3.42 ± 1.27‐fold enhancement; Figure S15, Supporting Information). Furthermore, we investigated the binding details within cascade amplification complexes and found similar Bi‐NIR‐Fb_ALFA_ occupancy for dCas9‐16×ALFA and dCas9‐24×ALFA (7.85 ± 1.29 vs 8.78 ± 1.60; Figure 2k; Figure S13d, Supporting Information), indicating that 16 copies of ALFA fusion were already saturated.

By integrating cascade amplification with background suppression, BRIGHT achieved ≈6.26‐fold and ≈6.76‐fold higher SNR than SunTag and PP7‐PCP in telomere labeling (Figure 1b,d) and an average SNR of 211.23 ± 104.46 for repetitive Chr3Rep (Figure 2e,f). Most notably, BRIGHT represents a significant advance over previous imaging systems, enabling nearly‐free‐background tracking of non‐repetitive loci with an SNR up to 284.84—far exceeding existing methods (Figure 3e). The SNRs of Chr3Rep and MUC4.1 labeling were higher than telomeres (Figure 1c, 2e, and 3e), likely because the high telomere abundance caused insufficient NIR molecules allocation per telomere at fixed expression levels of BRIGHT components. Thus, BRIGHT is more suitable for labeling non‐telomeric targets.

Beyond SNR enhancement, BRIGHT features a streamlined three‐component design, including dCas9‐n×ALFA, Bi‐NIR‐Fb_ALFA_, and sgRNA. The ALFA/Nb_ALFA_ system was selected with three reasons.^[^ ^25^ ^]^ First, the 13‐amino‐acid ALFA tag (SRLEEELRRRLTE) is compact and ideal for cascade assemblies, minimizing component size to avoid interference with target movement. Second, ALFA is an artificial sequence absent from the human proteome, avoiding nonspecific interactions with endogenous cellular components. Third, Nb_ALFA_ binds ALFA with ultrahigh affinity (*K_d_* = 26 pM), contributing to the exceptional stability of the BRIGHT complex as demonstrated by FRAP. Among the three components, Bi‐NIR‐Fb_ALFA_ is the key multifunctional element, simplifying the design by integrating oligomerization domains, fluorescent reporters, and specific binding modules, while supporting features such as background denoising, thus outperforming previous cascade systems.^[^ ^17^, ^18^ ^]^

BRIGHT also exhibits flexible modularity, enabling the substitution of any of its three components. For instance, dCas9 variants from different bacterial species could be adopted. By replacing the FPs inserted in Bi‐Nb_ALFA_, the fluorescence color of BRIGHT can be tuned. Additionally, alternative tag/Nb pairs, such as BC2‐tag/bivBC2‐Nb,^[^ ^64^ ^]^ can also be employed. Furthermore, the combination of diverse dCas9 variants, FPs, and tag/Nb systems will enable orthogonal imaging, facilitating investigations into multiple non‐repeated genomic loci within one cell.

We applied BRIGHT to study chromatin dynamics, the relationship between nuclear localization and transcriptional regulation, and eccDNA heterogeneity. We verified that BRIGHT showed consistency in Chr3Rep dynamic tracking with established systems and didn't perturb native molecular behavior (Figure 5c). The high SNR of BRIGHT permits accurate nuclear location and dynamic tracking of interested targets, promoting studies in 3D nuclear organization. Since eccDNA shares identical sequences with the genome except at the unique junction, CRISPR array‐based methods are not applicable. BRIGHT overcomes this limitation by targeting the junction sequence using only a single sgRNA. Nonetheless, the observed heterogeneity in copy number and motion patterns remains unlinked to functional outcomes, such as intracellular variability in oncogene expression, tumor adaptive evolution, clonal selection, and drug resistance,^[^ ^65^, ^66^ ^]^ which could be further addressed by integrating BRIGHT imaging with complementary approaches.

Although BRIGHT enables high‐SNR labeling and broad applicability, it has several limitations. First, Bi‐NIR‐Fb_ALFA_ may potentially crosslink free dCas9‐n×ALFA independently of DNA targets, producing unspecific NIR condensates at high component concentrations (Figures S2d and S3d, Supporting Information), thus maintaining relatively low expression is critical for locus‐specificity imaging. Second, although BRIGHT visualizes a non‐repetitive sequence with a single sgRNA, but off‐target binding combined with cascade amplification may cause nonspecific labeling, a common issue in single sgRNA‐based systems.^[^ ^19^ ^]^ This can be alleviated by selecting targets with minimal genomic similarity or using dCas9 variants with stricter protospacer adjacent motif (PAM) requirements. Third, its potential long‐term effects on cellular function require further investigation, including long‐term phototoxicity‐related impacts on mitochondrial activity, gene expression, and cell viability or proliferation,^[^ ^67^ ^]^ as well as prolonged CRISPR/dCas9‐based binding which may impede DNA replication, alter chromatin accessibility, and induce cellular stress from exogenous protein expression.^[^ ^68^ ^]^

Overall, the design concept of BRIGHT offers a new framework for advancing CRISPR‐based imaging tools, holding great promise to drive 4D nucleome studies.^[^ ^1^ ^]^ Furthermore, it can be extended to RNA imaging, antiviral drug screening, and in vivo imaging, leveraging the deep tissue penetration of NIR fluorophores.

## 4 Experimental Section

### Plasmid Construction

Plasmids of the dCas9‐SunTag system, originally obtained from Addgene, were kind gifts from Prof. Yu Zhang's lab at the National Institute of Biological Sciences, Beijing,^[^ ^69^ ^]^ including pHRdSV40‐NLS‐dCas9‐24×GCN4_v4‐NLS‐P2A‐BFP‐dWPRE (Addgene #60910) and pHR‐scFv‐GCN4‐sfGFP‐GB1‐NLS‐dWPRE (Addgene #60907). The pSLQ1651‐sgTelomere(F+E) plasmid was purchased from Ke Lei Biotechnology Co., Ltd (KZ1031). Plasmids of the PP7‐PCP system were obtained from MiaoLingPlasmid, China, including pHAGE‐TO‐dCas9 (P0906), pHAGE‐EFS‐PCP‐3×GFPnls (P13678), and pLH‐sgRNA1‐2×PP7 (P13646). Plasmids of the BRIGHT system were listed in Table S1 (Supporting Information). Plasmids expressing dCas9‐n×ALFA were constructed by replacing 24×GCN4‐P2A‐TagBFP with an artificially synthesized n×ALFA‐P2A‐TagBFP fragment between the BamHI and SalI sites (Sangon Biotech (Shanghai) Co., Ltd.). Plasmids expressing Bi‐NIR‐Fb_ALFA_, Tri‐NIR‐Fb_ALFA_, and Bi‐miniGFP‐Fb_ALFA_ were constructed by inserting artificially synthesized Bi‐NIR‐Fb_ALFA_‐NLS, Tri‐NIR‐Fb_ALFA_‐NLS, and Bi‐miniGFP‐Fb_ALFA_‐NLS fragments into the pcDNA3.1(+) vector between the KpnI and BamHI sites, with NIR‐Fb_ALFA_ copies connected via (G_4_S)_5_ linker. Plasmids expressing antigen‐dependent Mo‐NIR‐Fb_ALFA_, “always‐on” Mo‐NIR‐Fb_ALFA_, or “always‐on” Bi‐NIR‐Fb_ALFA_ were subcloned into the pcDNA3.1(+) backbone using the Yieasen Gibson assembly kit (10923ES50). The EF‐1α core promoter and hPGK promoter were PCR‐amplified from pHAGE‐EFS‐PCP‐3×GFPnls (P13678) and pLH‐sgRNA1‐2×PP7 (P13646), respectively. A 39‐bp minimal CMV promoter was prepared by annealing single‐stranded complementary oligos (PCR primers and oligos were listed in Table S2, Supporting Information). These promoters were inserted between the MluI and SacI sites to replace CMV enhancer and CMV promoter in the pcDNA3.1(+) vector. Plasmid expressing the GFP‐miRFP670nano3 fusion protein was constructed by PCR amplification from pHAGE‐EFS‐PCP‐3×GFPnls and antigen‐dependent Mo‐NIR‐Fb_ALFA_ (PCR primers were listed in Table S2, Supporting Information). Plasmids of the St1 dCas9 system were gifts from Thoru Pederson, including pHAGE‐TO‐nls‐st1dCas9‐3nls‐3×GFP‐2nls (Addgene #64113) and pLH‐stsgRNA3.1 (Addgene #64118).^[^ ^9^ ^]^ SpCas9‐related sgRNAs used in this study were generated with *mCherry* gene truncated as previously described.^[^ ^70^ ^]^ SpCas9‐related 2×sgRNAs and St1Cas9‐related sgRNAs were constructed by inserting guide sequences into the sgRNA backbone between two BbsI sites to replace the CcdB gene.^[^ ^9^ ^]^

### Cell Culture

HEK293T cells (RRID, CVCL_0063, Procell #CL‐0005) were kindly provided by Wuhan Pricella Biotechnology Co., Ltd., and were verified to be free of contamination before delivery. HepG2 cells (RRID, CVCL_0027, ATCC, HB‐8065) were originally purchased from ATCC and have been stably maintained in the laboratory under standard culture conditions, displaying consistent morphology and growth characteristics. Both cell lines were cultured in Dulbecco's Modified Eagle's Medium (DMEM) (Vivacell #C3113‐0500) with 10% fetal bovine serum (FBS) (Vivacell #C04001‐500), and 1% penicillin‐streptomycin (Gibco, ThermoFisher Scientific #15140122). HeLa cells (RRID, CVCL_0030, ATCC, CCL‐2) were also originally purchased from ATCC and were stably maintained in the laboratory with healthy morphology and growth characteristics. HeLa cells were cultured in Eagle's Minimum Essential Medium (EMEM) (WISENT #320‐006‐CL) with 10% FBS, and 1% penicillin‐streptomycin. U2OS cells (RRID, CVCL_0042, Boster #CX0030) were purchased from the BOSTER Biological Technology Co., Ltd., and were verified to be free of contamination before delivery. U2OS cells were cultured in McCoy's 5A medium (Boster #PYG0026) with 10% FBS, and 1% penicillin‐streptomycin. Cells were maintained at 37 °C and 5% CO_2_ humidified incubator.

### Cell Transfection

Cells were seeded in 35 mm glass‐bottom dishes with 20 mm micro‐wells (Cellvis #D35‐20‐1.5‐N) at a density of 150 000 (HEK293T) or 10 000 (HeLa, HepG2 and U2OS) cells/well 24 h before transfection. Transfections were performed using Lipofectamine 3000 (Invitrogen #L3000015) or jetPRIME (Polyplus #101000046) in Opti‐MEM medium (Gibico #31985070) according to the manufacturer's instructions. For regular CRISPR‐SunTag transfection, 1250 ng of dCas9‐GCN4, 500 ng of scFv‐sfGFP, and 750 ng of sgRNA were used. For regular PP7‐PCP transfection, 500 ng of dCas9, 200 ng of PCP‐3×GFP, and 800 ng of 2×PP7‐sgRNA were used. For regular BRIGHT transfection, 500 ng of dCas9‐n×ALFA, 500 ng of nanobody, and 800 ng of sgRNA were used. For regular St1 dCas9 transfection, 50 ng of St1 dCas9‐3×GFP and 250 ng of St1 sgRNA were used. Following transfection, cells were incubated for an additional 12–24 h before imaging. Hoechst 33342 (Yeasen #40732ES03) was used at a final concentration of 1 µg mL^−1^ to stain the nuclei.

### Fluorescence Microscopy

Live‐cell imaging was conducted on a Nikon Ti2‐E inverted microscope, equipped with a Nikon A1R HD25 confocal scanning system and controlled by NIS‐Elements AR software. The system included a 20× air objective (CFI Plan Apo λ 20×, NA 0.75), a 60× oil‐immersion objective (CFI Plan Apo λ 60×, NA 1.4), a 100× oil‐immersion objective (Plan Apo VC 100× Oil, NA 1.45), a Nikon A1 large field of view (LFOV) detector, and a Perfect Focus System (PFS). Time‐lapse imaging was performed at 37 °C and 5% CO_2_, with PFS providing real‐time automated focus correction. TagBFP and Hoechst were detected using a 405‐nm laser and 450/50‐nm filter cube. GFP, sfGFP, and miniGFP were detected using a 488‐nm laser and 525/50‐nm filter cube. Cy3 and mCherry were detected using a 561‐nm laser and 595/50‐nm filter cube. miRFP670nano3 were detected using a 640‐nm laser and 700/75‐nm filter cube. Z‐stacks were collected at a step size of 0.2–0.5 µm to image the entire nucleus.

### Imaging Processing and Analysis

For SNR calculation, fluorescence intensity measurements were performed using Fiji software as previously described.^[^ ^71^ ^]^ Multiple BRIGHT puncta were manually segmented and outlined to measure their intensities. To determine the background intensity, identical circles were randomly placed within the nucleus but outside the puncta. Pixel values within these background circles were extracted via Macros plugins implemented in Fiji, and the mean background intensity along with its standard deviation was calculated accordingly. The SNR was calculated using the following formula:^[^ ^20^, ^22^ ^]^

(1) $$\begin{array}{ccc} \mathrm{SNR}\; & = & \frac{P_{\mathrm{signal}}}{P_{\mathrm{background}}}\\ & & =\frac{\mathrm{Maximum}\;\mathrm{intensity}\;\mathrm{of}\;\mathrm{spots}\;\mathrm{signal}-\mathrm{mean}\;\mathrm{intensity}\;\mathrm{of}\;\mathrm{background}}{\mathrm{std}.\mathrm{dev}.\mathrm{of}\;\mathrm{background}} \end{array}$$

All the plots were graphed in Origin 2024. For Z‐stack processing, image stacks acquired at defined step sizes were processed using NIS‐Elements AR or Fiji to generate maximal intensity projection (MIP) and reconstructed XYZ‐3D images. Unless otherwise noted, all fluorescence images shown were MIP representations. Co‐localization analysis between different channels was performed by Merge function in Fiji. Plot‐profile quantification was conducted by Line Tool and Plot Profile function in Fiji, and the exported data were normalized to facilitate comparison across between different channels.

### Fluorescence Recovery After Photobleaching (FRAP)

FRAP experiments were performed 24 h after transfection of HEK293T cells with BRIGHT components targeting Chr3Rep, using a Nikon Ti2‐E inverted microscope equipped with an A1R HD25 confocal scanning system (Galvano scanner), a PMT detector, a 100× oil‐immersion objective (Plan Apo VC 100× Oil, NA 1.45). Data acquisition was performed with NIS‐Elements AR software. Specific BRIGHT puncta were selected as regions of interest (ROIs), while an additional BRIGHT dot within the same field was designated as a reference, and nuclear background fluorescence intensity was recorded. A three‐phase time schedule was implemented. During the pre‐bleach phase, ROIs were imaged every 3 s for a total of 6 s to record the baseline fluorescence intensity, using a 640‐nm laser at 3% power. Photobleaching was then performed using the 640‐nm laser at 33% power for 0.12–0.24 s, reducing fluorescence to ≈30% of the baseline level.^[^ ^36^ ^]^ During the post‐bleach phase, fluorescence recovery was monitored by imaging ROIs every 3 s for 180 s, with the 640‐nm laser at 3% power.^[^ ^18^, ^35^ ^]^ The raw data were processed using EasyFRAP‐web for rapid FRAP dataset analysis,^[^ ^72^ ^]^ with double normalization recommended against both the reference and nuclear background. The final FRAP recovery curve was plotted using Origin 2024.

### RNA Isolation and RT‐qPCR

After cells were transfected as described in the Cell transfection section for 24 h, total RNA was extracted from cells using E.Z.N.A. Total RNA Kit II (Omega #R6934) according to the manufacturer's instructions, and its concentration was measured with a spectrophotometer. Single‐stranded cDNA was synthesized by reverse transcription with oligo(dT) primers. RT‐qPCR was performed using POWRUP SYBR MASTER MIX (Thermo Fisher #A25742) in ABI 7500. All data were collected and normalized using β‐actin mRNA as the internal reference. Primer sequences for RT‐qPCR are listed in Table S2 (Supporting Information).

### Oligo‐FISH

To validate the labeling specificity of MUC4.1 puncta, oligo‐FISH was performed following BRIGHT imaging. After removing the culture medium and washing once with PBS, cells were fixed with 4% paraformaldehyde for 15 min at room temperature (RT). Then cells were washed three times with PBS (5 min/each) and permeabilized with 0.7% Triton X‐100 in 2×SSC for 30 min at RT. After two additional PBS washes, cells were incubated with 100 µg/mL RNase A at 37°C for 1 h, followed by two washes with 2×SSC and a brief incubation in PBS for 5 min. Next, cells underwent gradient dehydration using 70%, 85%, and 100% ethanol. Once air‐dried, they were denatured in 70% formamide/2×SSC at 80°C for 5 min, followed by incubation in pre‐chilled (−20°C) 70%, 85%, and 100% ethanol on ice. Cells were air dried, and 2 ng mL^−1^ Cy3‐labeled M4‐E2Rep oligo‐FISH probes (/Cy3/CTTCCTGTCACCGAC) in hybridizing solution (2×SSC containing 10% dextran sulfate, 50% formamide, and 500 ng mL^−1^ salmon sperm DNA) were added, and the cells were sealed for incubation at 37°C. After 16 h of hybridization, cells were washed three times with 2×SSC and counterstained with DAPI for further observation.

### Single‐Particle Tracking Analysis

Images were taken with a Multi‐SIM (Naxin Optoelectronics Co., Ltd., Beijing, China), spaced by 200 ms with a total of 50 shots. Chr3Rep image stacks were first detected by TrackMate plugins in Fiji. Fluorescent puncta were identified in each frame by using Laplacian of Gaussian (LoG) detector with a suitable estimated object diameter of 5 camera pixels and a quality threshold. Then, a simple LAP tracker for tracking was used. The nearest neighbors identified puncta within a maximum distance range of 5 camera pixels in the previous frame. The gap larger than 5 consecutive frames was treated as two particles. The data were exported from Fiji and analyzed by MATLAB tracking package “msdanalyzer.”^[^ ^73^ ^]^ All MSD curves of target sites were obtained from at least 20 cells, and averaged for display. The MSD curves were fitted by:^[^ ^19^ ^]^

(2) $$\mathrm{MSD}\left(t\right)=A\left(1-e^{-t/\tau }\right)+4D_{eff}t+v^{2}t^{2}$$

where *A* was the confinement area, *τ* was a constant, *D_eff_* was the macroscopic diffusion coefficient, and *v* was the velocity of active transport.^[^ ^74^ ^]^ And *D_eff_* was calculated by *D_eff_* = *A*/4*τ*.

### Nuclear Localization Analysis

The distance of each spot to the nuclear periphery has been calculated as *L* using Fiji as the shortest distance from the spot to the nuclear membrane. The cell centroid was identified using Fiji, and the distance from the centroid to the nuclear membrane was calculated across the analyzed signal spot as *D*. The ratio of *L* to *D* was used to evaluate the relative proximity of the signal spot to the cell membrane.

### Estimation the Number of DNA Signal Foci

To estimate the number of DNA signal foci, a MIP of the Z‐stack was first generated using NIS‐Elements AR software. The resulting 2D image was imported into Fiji and converted to 8‐bit format. A suitable threshold was then applied to isolate bright signal spots from the background. Following thresholding, binary images were obtained, and the Watershed function was applied to separate closely adjacent or partially overlapping signals. Finally, the Analyze Particles function was used to count individual foci, with the particle size range adjusted to exclude background noise. Only signals fully contained within the ROIs were included in the final count.

### Extraction, amplification, Sequencing, and Imaging of eccDNA

To extract total eccDNA, 5 × 10^6^ HepG2 cells were used to harvest the whole genome DNA according to the manufacturer's instructions (Tiangen #DP304‐03). Plasmid‐Safe ATP‐dependent DNase (Lucigen #E3110K) was used to digest the remaining linear DNA after eluting column‐bound DNA at 37 °C for 4 h, and the DNase was heat‐inactivated for 30 min at 70°C. Then, three rounds of PCR were conducted using primers listed in Table S2 (Supporting Information), followed by Sanger sequencing. The junction sequence used for BRIGHT imaging of eccBEND3 is listed in Table S3 (Supporting Information).

### Phototoxicity Experiments

ROS levels were measured 24 h after transfection of the BRIGHT system in HEK293T cells. Cells were incubated with 10 µm 2′,7′‐dichlorodihydrofluorescein diacetate (DCFH‐DA) (Sigma, #D6883) in DMEM with 10% FBS at 37°C for 5 min, washed three times with PBS, and imaged on a Nikon Ti2‐E inverted microscope equipped with a 100× oil‐immersion objective (Plan Apo VC 100× Oil, NA 1.45) and a PFS. To mimic the maximum exposure condition used in routine imaging, cells were illuminated with a 640‐nm laser at 10.0% laser power, with PMT high voltage set to 120 and PMT offset to 0. Cells with BRIGHT puncta were repeatedly excited in a time series of single optical sections (15 cycles at 5 s intervals), while ROS‐associated DCFH‐DA green fluorescence was simultaneously recorded using a 488‐nm laser. A negative control group of non‐BRIGHT transfected cells without 640‐nm illumination was included and stained with DCFH‐DA and monitored by a 488‐nm laser to establish the baseline ROS level. For analysis, the increase in ROS was quantified as the percentage change in fluorescence intensity of each frame relative to the first frame.^[^ ^33^ ^]^

### Statistical Analysis

All statistical data were performed at least three replicates, and all representative images shown were obtained from experiments independently repeated three times with similar results. Statistical analyses were performed using Excel and Origin 2024. Statistical comparisons were made using two‐tailed unpaired Student's *t*‐test, Mann‐Whitney test, or Kruskal‐Wallis test. For all figures, a *p* value > 0.05 was considered no significance (n.s.), and significance levels were denoted as follows, ^*^ *p* < 0.05, ^**^ *p* < 0.01, ^***^ *p* < 0.001, and ^****^ *p* < 0.0001.

## Conflict of Interest

The authors declare no conflict of interest.

## Supporting information



Supporting Information

Supporting Information

Supplemental Video 1

Supplemental Video 2

Supplemental Video 3

Supplemental Video 4

Supplemental Video 5

Supplemental Video 6

## Data Availability

The data that support the findings of this study are available from the corresponding author upon reasonable request.
