# Supplementary material for: BRIGHT Enables High‐SNR Live‐Cell Imaging of Non‐Repetitive Sequences via Bivalent Fluorescent Nanobody‐Mediated Cascade‐Dependent Illumination
Source: Adv Sci (Weinh). 2025 Oct 17;13(2):e13014. doi: 10.1002/advs.202513014 (PMC12786294; doi:10.1002/advs.202513014)
Supplement: Supplementary file 1 — Supporting Information [file ADVS-13-e13014-s005.docx]

**Title**

BRIGHT Enables High-SNR Live-Cell Imaging of Non-repetitive Sequences via Bivalent Fluorescent Nanobody-Mediated Cascade-Dependent Illumination

*Lei Feng, Tao Huang, Yanxi Han, Meng Tian, Duo Wang, Zilong Mei, Yu Ma, Yingshuo Ma, Jinming Li^*^ and Rui Zhang^*^*


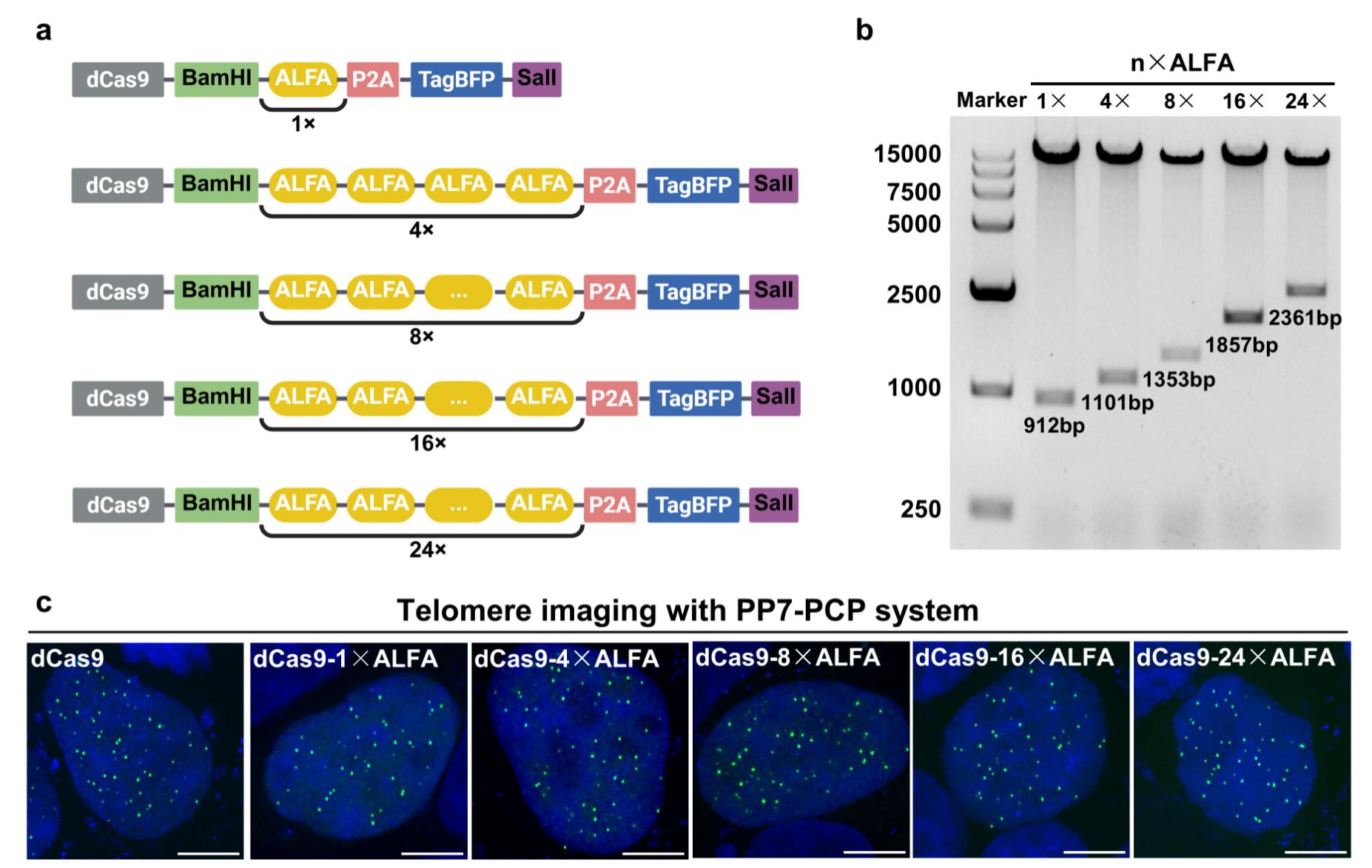


**Figure S1.** **Construction and functional validation of dCas9-n×ALFA fusion proteins.**

**(a)** Schematic design of dCas9-n×ALFA (n = 1, 4, 8, 16, 24) fusion protein expression constructs. Artificially synthesized sequences encoding n×ALFA, P2A, and TagBFP are inserted between BamHI and SalI restriction sites. Created in BioRender. Zhang, R. (2025) https://BioRender.com/8wd7z40. **(b)** Gel electrophoresis analysis of BamHI and SalI double-digested plasmids to verify the correct sequence length of the inserted ALFA tandem repeats. **(c)** Representative images of telomere labeling with PP7-PCP system. dCas9-based labeling served as a positive control to validate the binding capacity of dCas9-n×ALFA. The results indicate that ALFA fusion don’t impair the telomere targeting ability of dCas9. Scale bar, 5 μm.


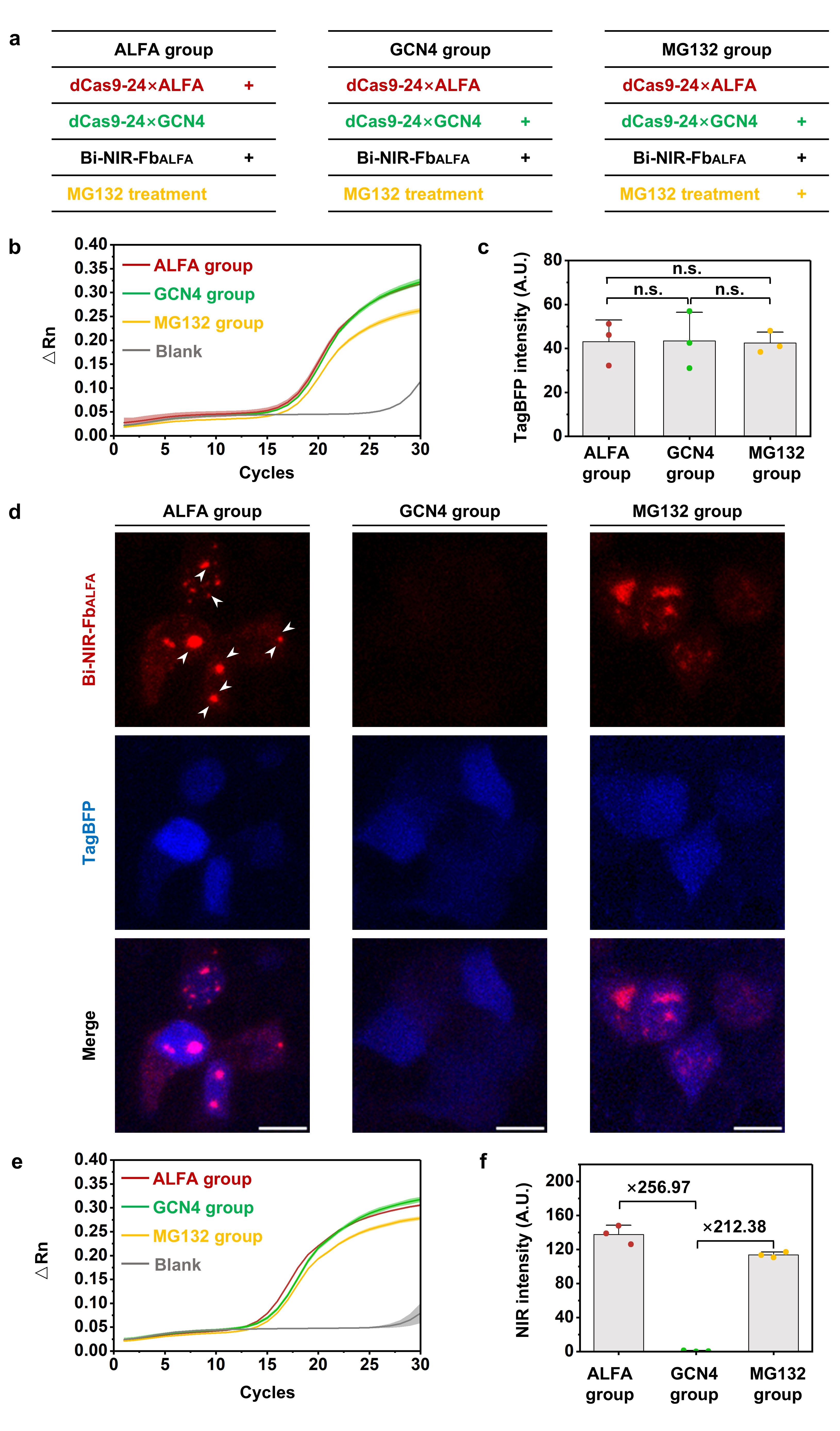


**Figure S2. Bi-NIR-Fb_ALFA_ exhibiting ALFA-dependent fluorescence and proteasomal degradation.**

**(a)** Description of three co-transfection groups: the ALFA group (0.3 pmol dCas9-24×ALFA and 0.15 pmol Bi-NIR-Fb_ALFA_), the GCN4 group (0.3 pmol dCas9-24×GCN4 and 0.15 pmol Bi-NIR-Fb_ALFA_), and the MG132 group (GCN4 group with10 μM MG132 treatment for 6 h after 16 h transfection). **(b)** RT-qPCR analysis showing comparable mRNA expression levels of dCas9-24×ALFA and dCas9-24×GCN4 in all three groups. Blank means HEK293T cells without transfection. **(c)** Quantification of TagBFP fluorescence intensity revealing similar protein expression levels of dCas9-24×ALFA and dCas9-24×GCN4 across groups. TagBFP was used to indicate the expression of dCas9-24×ALFA and dCas9-24×GCN4. Statistical analysis was performed using the Kruskal-Wallis test. *p* > 0.05. **(d)** Representative fluorescence images of the three con-transfection groups. Scale bar, 20 μm. White arrows indicate nuclear NIR condensates that could interfere with signal interpretation due to CMV promoter-driven overexpression of Bi-NIR-Fb_ALFA_. **(e)** RT-qPCR analysis showing Bi-NIR-Fb_ALFA_ mRNA expression across all groups. Blank means HEK293T cells without transfection. **(f)** Quantification of NIR fluorescence intensity in the three groups. Despite similar Bi-NIR-Fb_ALFA_ mRNA levels, strong NIR fluorescence is observed in the ALFA group. Upon MG132 treatment, NIR fluorescence restored to 82.65% of the ALFA group. Data in **(c)** and **(f)** are presented as the means ± SD; *n* = 3 independent replicates; individual data points are shown as colored dots.


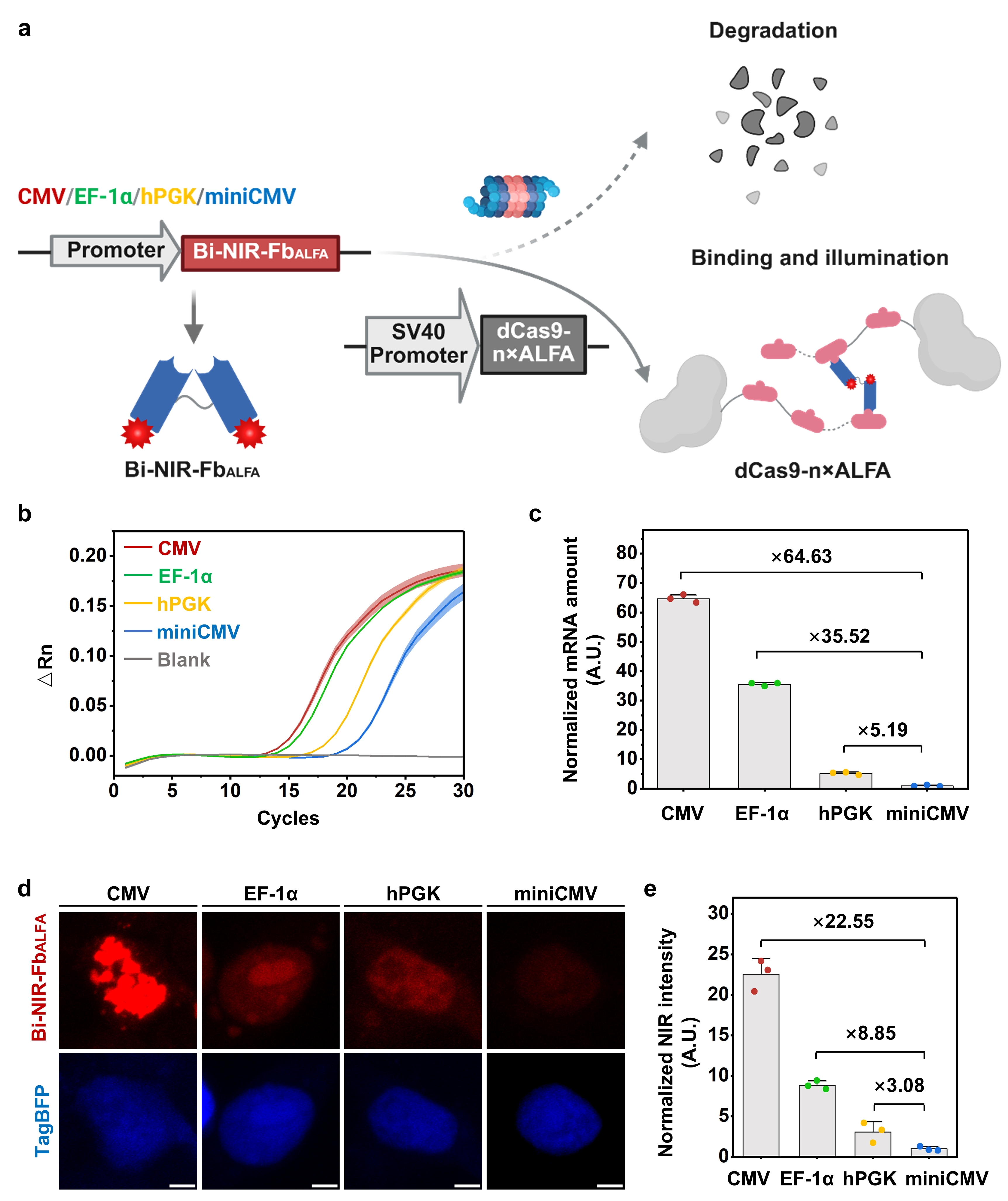


**Figure S3. The hPGK promoter identified as optimal for Bi-NIR-Fb_ALFA_ expression regulation.**

**(a)** Schematic of promoter optimization strategy for proper Bi-NIR-Fb_ALFA_ expression. Created in BioRender. Zhang, R. (2025) https://BioRender.com/w2sqw83. **(b)** RT-qPCR analysis showing that the CMV promoter drives the highest mRNA level, followed by the EF-1α, hPGK, and miniCMV promoters. Blank means HEK293T cells without transfection. **(c)** Normalized mRNA expression levels of Bi-NIR-Fb_ALFA_ under four different promoters. Expression levels were normalized to β-actin as an internal control and then to the miniCMV group for comparison. **(d)** Representative fluorescence images of the four groups. CMV-driven expression leads to prominent NIR condensates, EF-1α causes nonspecific nucleolar localization, and miniCMV results in too weak fluorescence for effective fluorescence detection. Scale bar, 5 μm. **(e)** NIR fluorescence intensity normalized to the miniCMV group for comparison across four different promoters. Data in **(c) and (e)** are presented as the means ± SD; *n* = 3 independent replicates; individual data points are shown as colored dots.


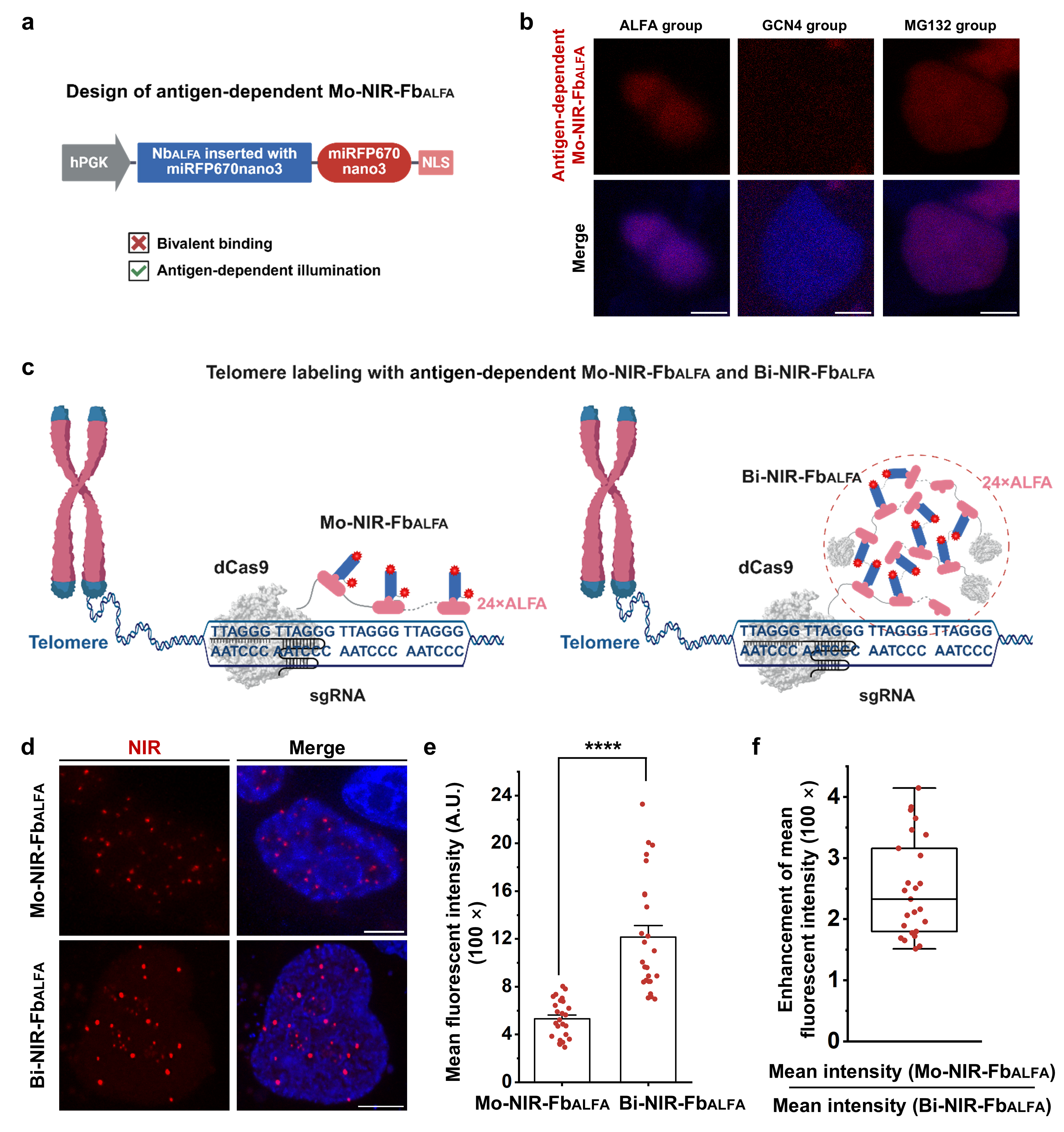


**Figure S4. Bi-NIR-Fb_ALFA_ enabling cascade enhancement of NIR fluorescence in telomere labeling through its bivalent binding ability.**

**(a)** Schematic showing the design of antigen-dependent Mo-NIR-Fb_ALFA_ carrying two miRFP670nano3 to control the same fluorophore stoichiometry with Bi-NIR-Fb_ALFA_. Created in BioRender. Zhang, R. (2025) https://BioRender.com/xhfr6tc. **(b)** Representative images of antigen-dependent Mo-NIR-Fb_ALFA_ co-expressed with dCas9-24×ALFA or dCas9-24×GCN4 (with or without MG132 treatment) in HEK293T cells. Scale bar, 5 μm. **(c)** Schematic of telomere labeling by antigen-dependent Mo-NIR-Fb_ALFA_ and antigen-dependent Bi-NIR-Fb_ALFA_ co-expressed with dCas9-24×ALFA. Protein structures were predicted with AlphaFold3^[1]^ and visualized in ChimeraX 1.9^[2]^. Remaining elements were created in BioRender. Zhang, R. (2025) https://BioRender.com/xhfr6tc. **(d)** Representative fluorescence images of telomere labeling by Mo-NIR-Fb_ALFA_ and Bi-NIR-Fb_ALFA_ in HEK293T cells. Scale bar, 5 μm. **(e)** Mean fluorescent intensity analysis of telomere imaging. Data are presented as means ± SEM for Mo-NIR-Fb_ALFA_ labeling (531.10 ± 31.88, *n* = 25 puncta) and Bi-NIR-Fb_ALFA_ labeling (1214.57 ± 96.66, *n* = 25 puncta). Statistical analysis was performed using the Mann-Whitney test. *p* < 0.0001. **(f)** Mean fluorescent intensity enhancement of Bi-NIR-Fb_ALFA_ labeling compared to Mo-NIR-Fb_ALFA_ labeling. Mean ± SD of SNR enhancement: 2.51 ± 0.81. Box plots show the median, and the whiskers extend to the minimum and maximum values. Image acquisition was performed under identical conditions for fluorescent intensity analysis.


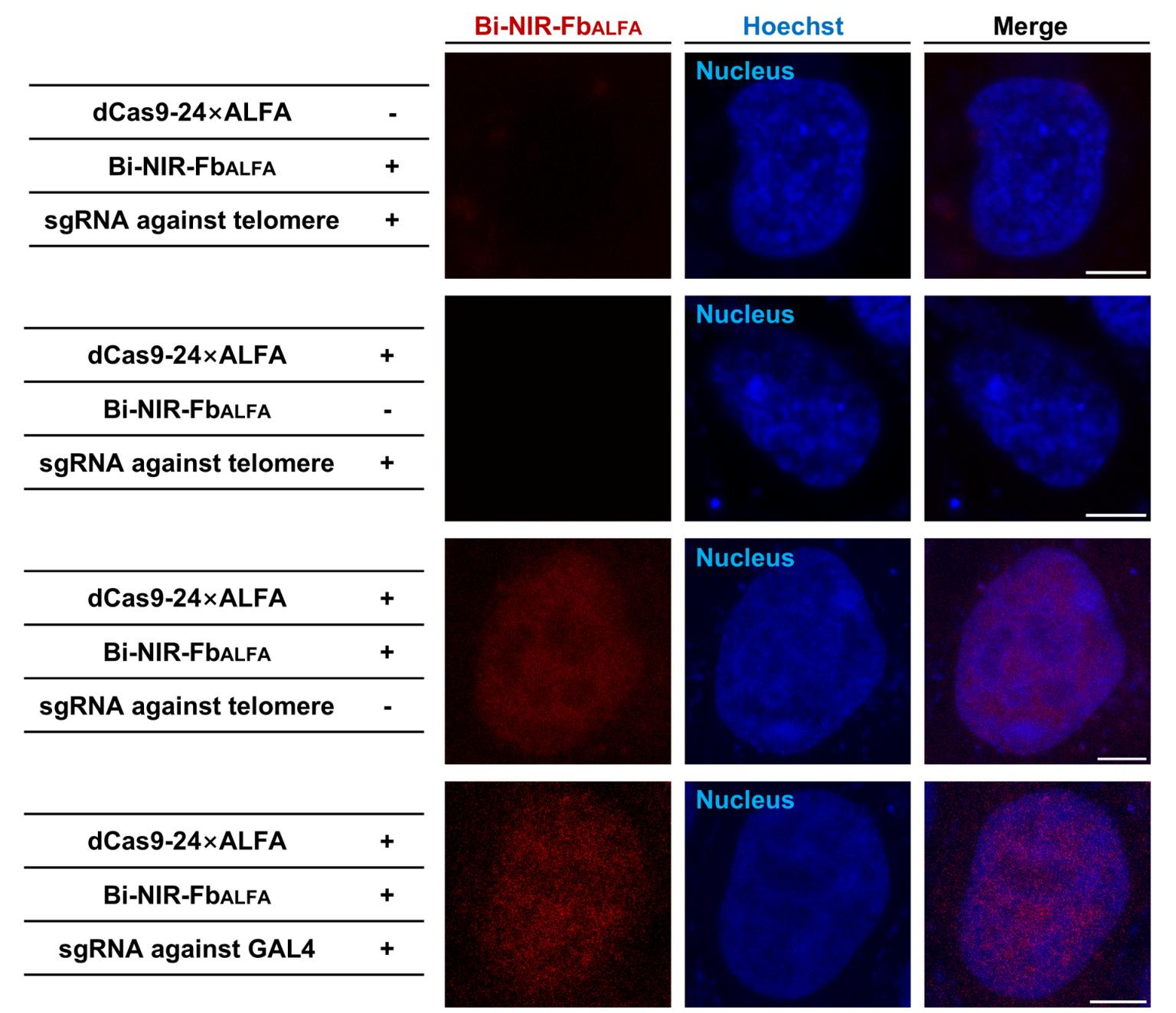


**Figure S5. Telomere imaging with various combinations of BRIGHT components.**

In the absence of dCas9-24×ALFA, almost no NIR fluorescence is detected, likely due to the degradation of Bi-NIR-Fb_ALFA_ without ALFA-mediated stabilization. The group lacking Bi-NIR-Fb_ALFA_ shows no NIR fluorescence, as the fluorescent element is missing. In the absence of sgRNA against telomere, NIR fluorescence is uniformly diffused throughout the nucleus, with no accumulation at the telomere site. These results demonstrate that all three BRIGHT components are required for the formation of distinct BRIGHT puncta. Cells transfected with sgGAL4 exhibits diffuse NIR fluorescence due to the absence of a corresponding target site in HEK293T cells, indicating the specificity of BRIGHT labeling. Scale bar, 5 μm.


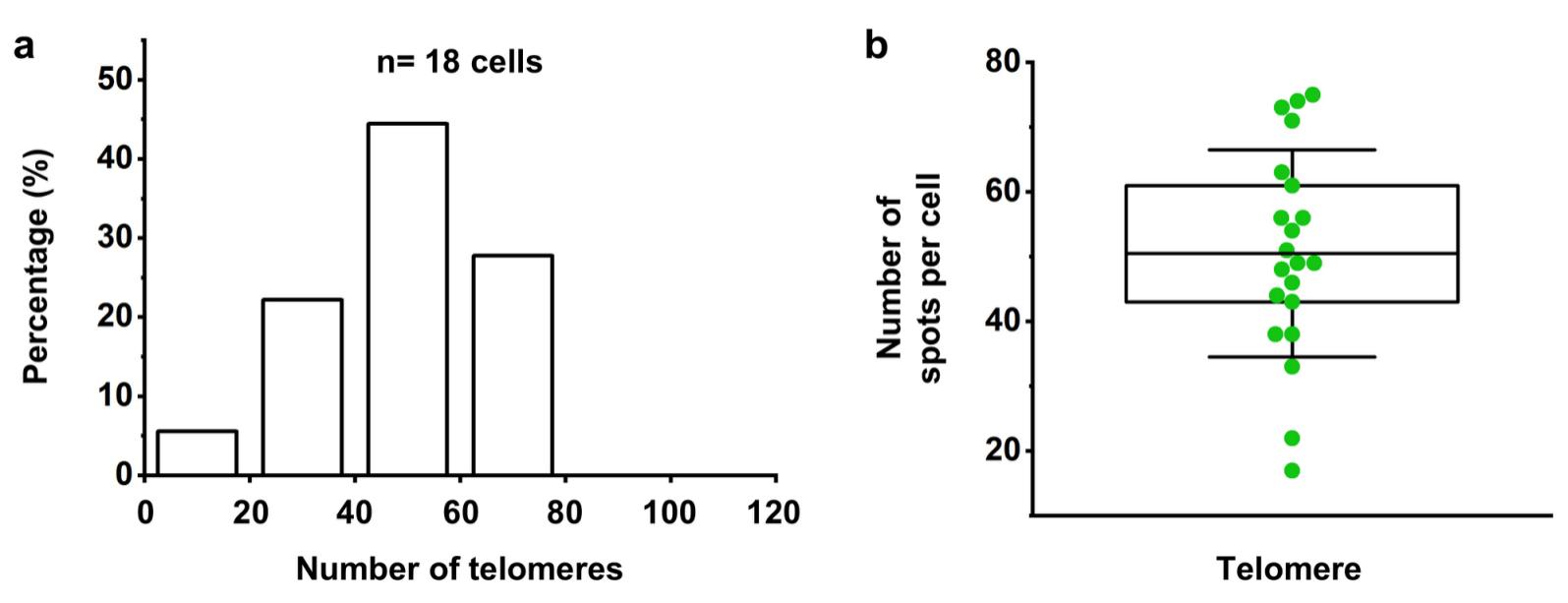


**Figure S6. Quantification of telomere puncta in HEK293T cells using the PP7-PCP system.**

**(a)** Histogram of telomere counts per single HEK293T cell obtained using PP7-PCP system (*n* = 18 cells). **(b)** Box plot showing the number of telomere puncta detected per cell using PP7-PCP system. Data are presented as mean ± SD (49.17 ± 16.21 puncta per cell, *n* = 18 cells), with individual data points shown as green dots.


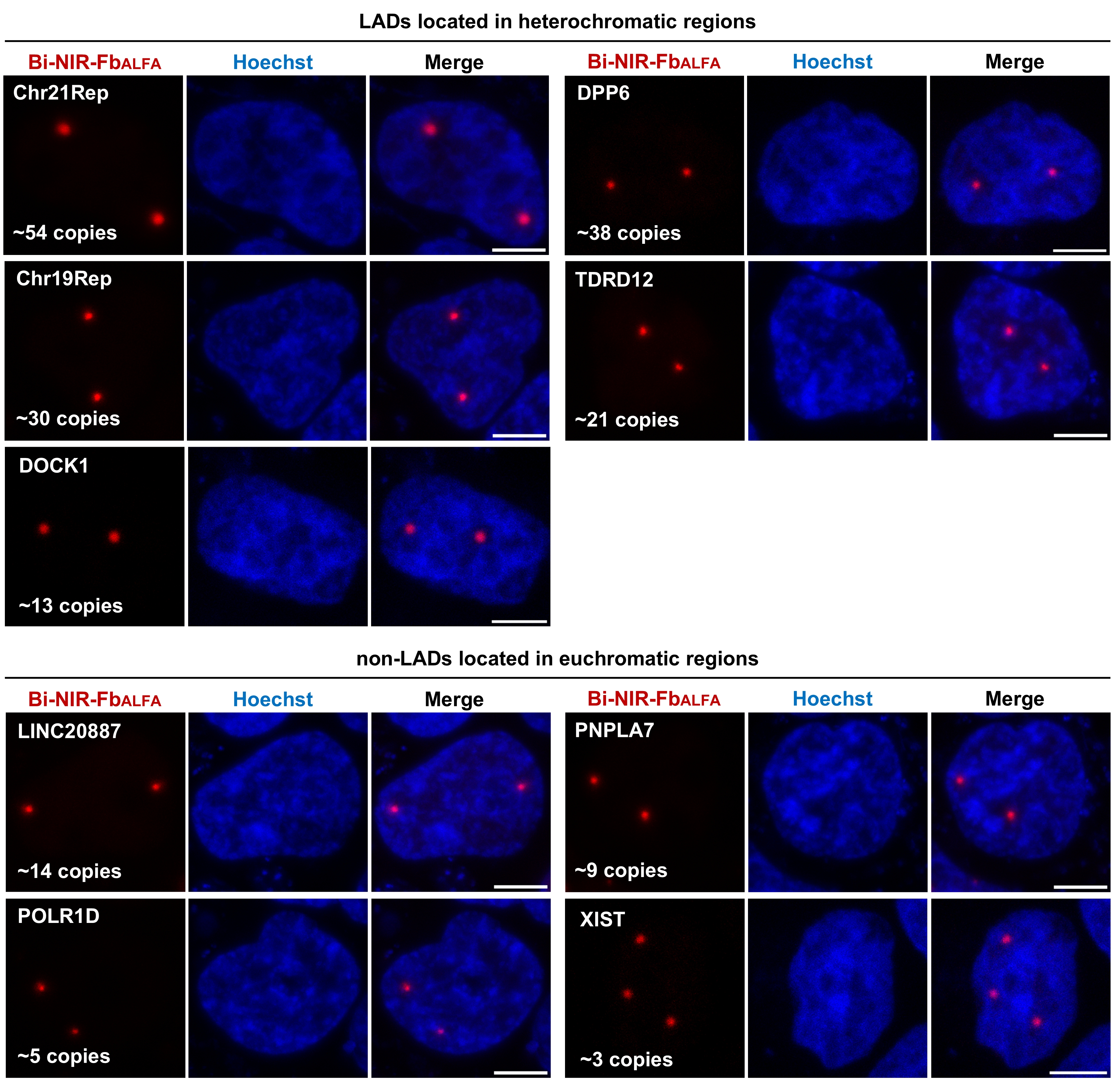


**Figure S7. Representative BRIGHT images of low-copy genomic loci ranging from 3 to 100 copies.**

BRIGHT towards low-copy genomic loci on chromosomes 7, 9, 10, 13, 17, 19, 21 and X. Among these loci, Chr21Rep, *DPP6*, Chr19Rep, *TDRD12* and *DOCK1* are LADs located in heterochromatic regions, whereas *LINC20887*, *PNPLA7*, *POLR1D*, and *XIST* are non-LADs locates in euchromatic regions. Scale bar, 5 μm.


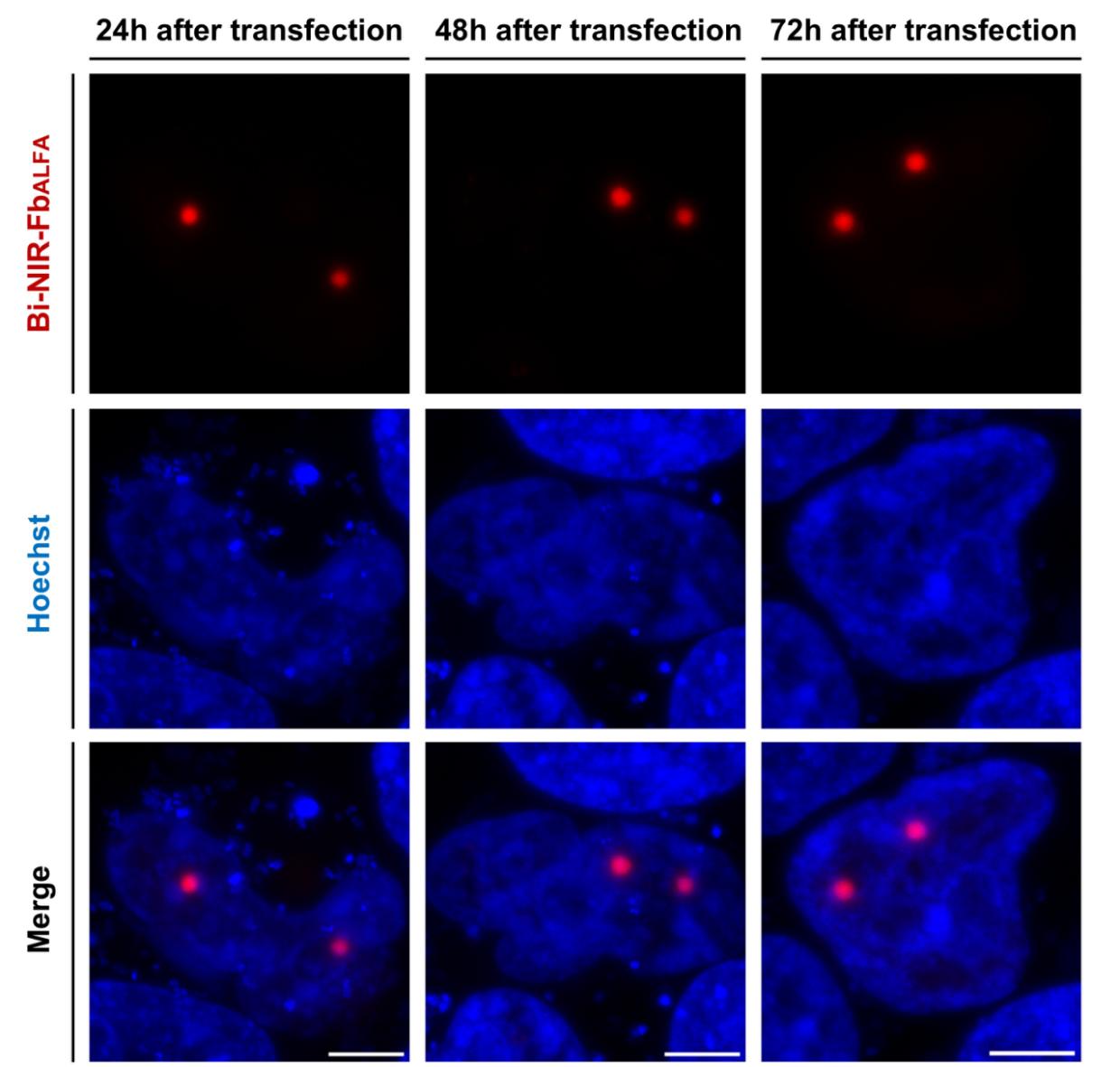


**Figure S8. Time-course Chr3Rep imaging up to 72 hours after transfection.**

Images were acquired at 24, 48, and 72 hours post-transfection. Scale bar, 5 μm.


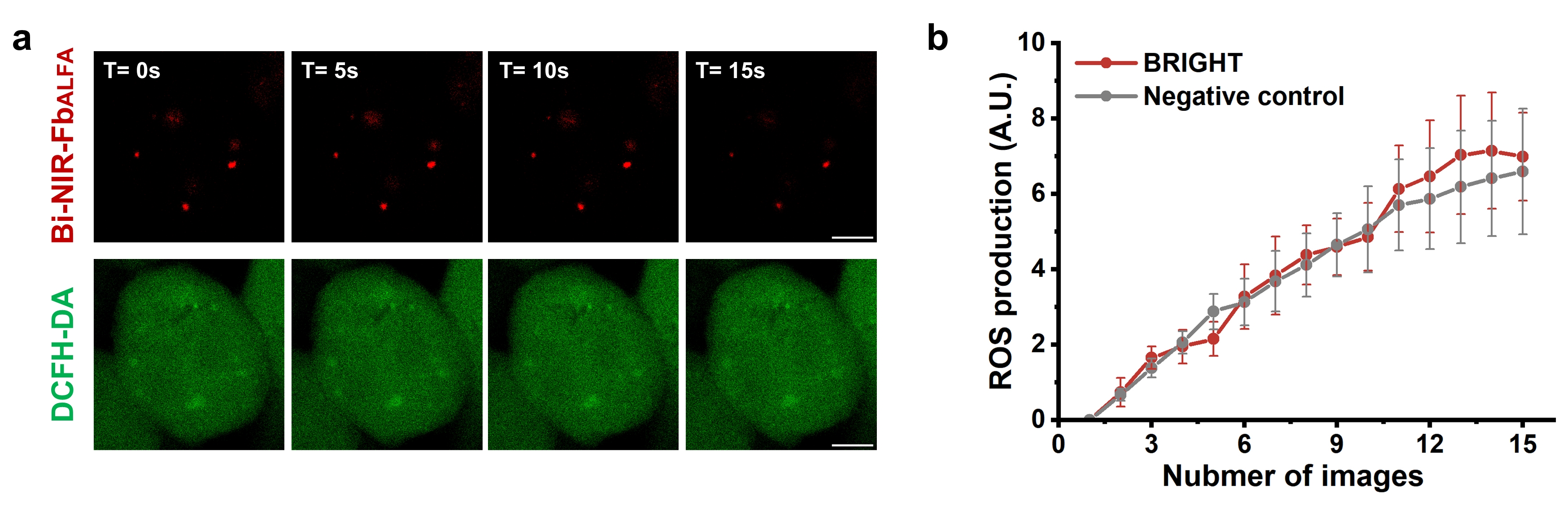


**Figure S9. BRIGHT labeling inducing low phototoxicity in HEK293T cells.**

**(a)** Representative images of ROS detection in BRIGHT-labeled cells targeting Chr3Rep. Scale bar, 5 μm. **(b)** Quantification showing that BRIGHT labeling caused low ROS increase compared with the baseline group. Data are presented as mean ± SEM (*n* = 5 cells per group).


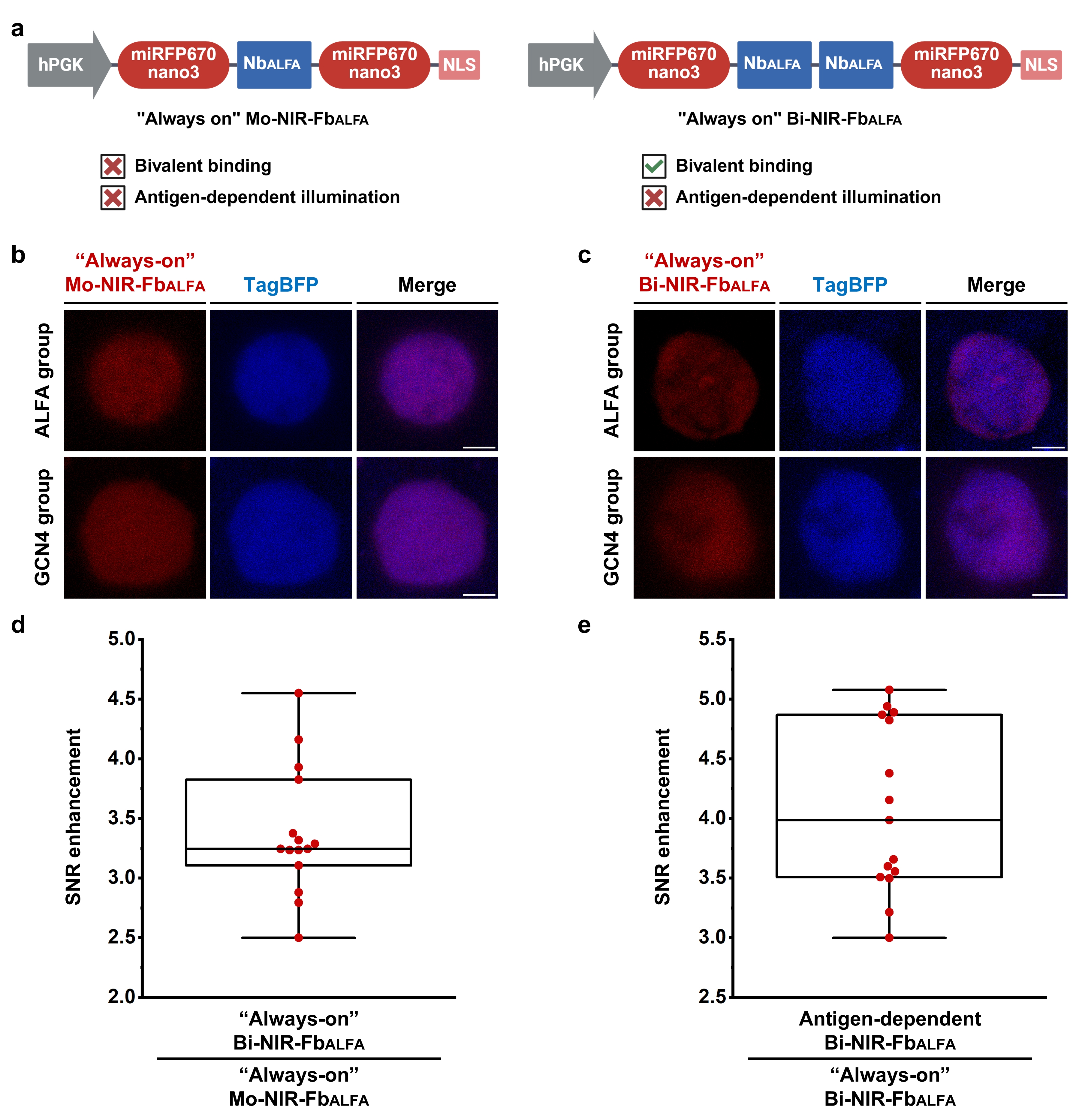


**Figure S10 Design and labeling of "always-on" Mo-NIR-Fb_ALFA_ and "always-on" Bi-NIR-Fb_ALFA._**

**(a)** Schematic showing the design of "always-on" Mo-NIR-Fb_ALFA_ and "always-on" Bi-NIR-Fb_ALFA_. "Always-on" Mo-NIR-Fb_ALFA_ was designed to carry two miRFP670nano3 to control the same fluorophore stoichiometry with "always-on" Bi-NIR-Fb_ALFA_. Created in BioRender. Zhang, R. (2025) https://BioRender.com/2ekkuyk. **(b)** Representative images of "always-on" Mo-NIR-Fb_ALFA_ co-expressed with dCas9-24×ALFA or dCas9-24×GCN4 in HEK293T cells. Scale bar, 5 μm. **(c)** Representative images of "always-on" Bi-NIR-Fb_ALFA_ co-expressed with dCas9-24×ALFA or dCas9-24×GCN4 in HEK293T cells. Scale bar, 5 μm. **(d)** SNR enhancement of Chr3Rep labeling with dCas9-24×ALFA and "always-on" Bi-NIR-Fb_ALFA_ compared to "always-on" Mo-NIR-Fb_ALFA_. Mean ± SD of SNR enhancement: 3.38 ± 0.54. Box plot shows the median, and the whiskers extend to the minimum and maximum values. **(e)** SNR enhancement of Chr3Rep labeling with dCas9-24×ALFA and antigen-dependent Bi-NIR-Fb_ALFA_ compared to "always-on" Bi-NIR-Fb_ALFA_. Mean ± SD of SNR enhancement: 4.08 ± 0.70. Box plot shows the median, and the whiskers extend to the minimum and maximum values.


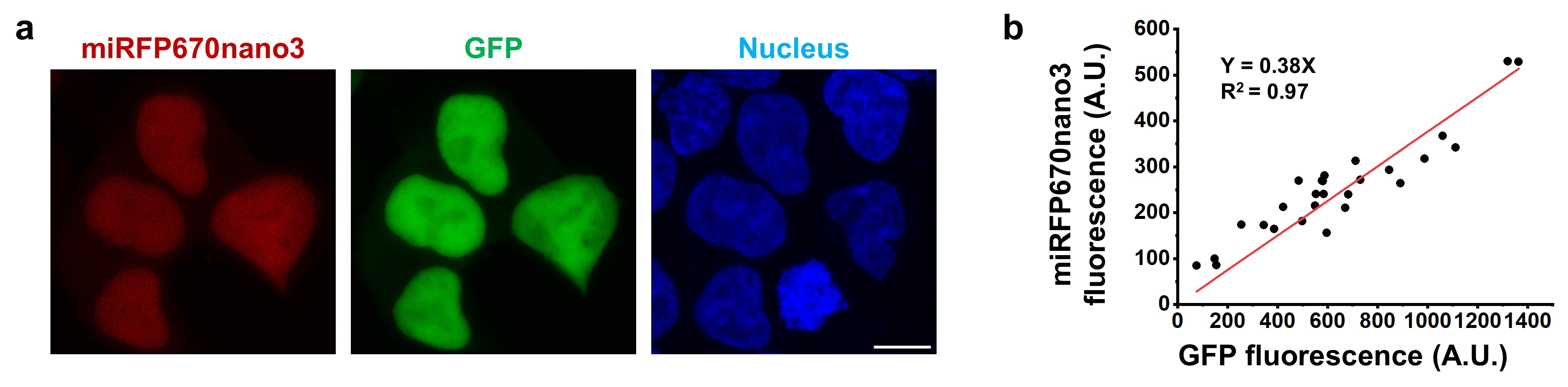


**Figure S11 Correction factor from the GFP-miRFP670nano3 fusion protein for Bi-NIR-Fb_ALFA_ occupancy analysis.**

**(a)** Representative images of the expression of GFP-miRFP670nano3 fusion protein in HEK293T cells, acquired 24 h after transfection. To correct for intrinsic intensity differences between the two FPs, we constructed a GFP-miRFP670nano3 fusion protein with the expression of miRFP670nano3 and GFP at a 1:1 ratio^[3]^ . Scale bar, 10 μm. **(b)** Scatter plot for correction factor calculation, yielding a value of 0.38. *n* = 27 cells. Each dot represents a single cell and the red line indicates the fitted line. Identical imaging conditions with Figure 2k were used for the calculations of the normalized Bi-NIR-Fb_ALFA_ occupancy.


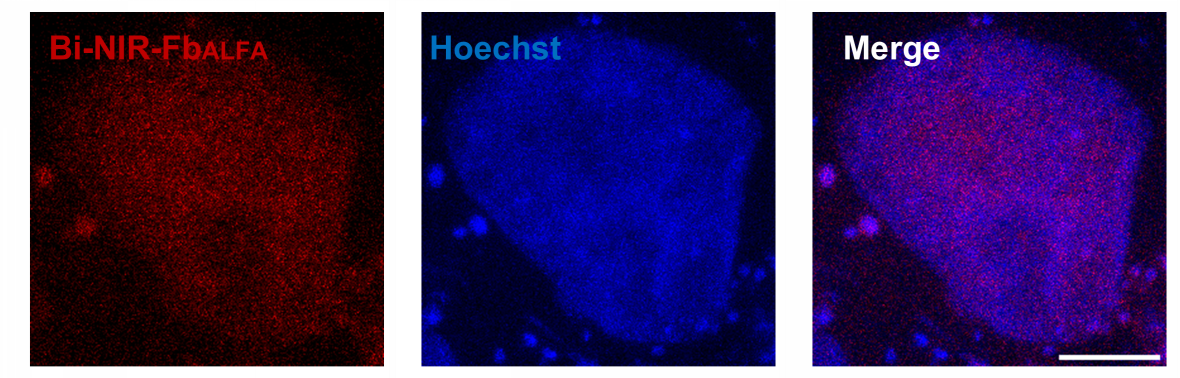


**Figure S12. Representative images of the negative control using sgGAL4.**

Representative images of BRIGHT labeling with sgGAL4. Scale bar, 5 μm.


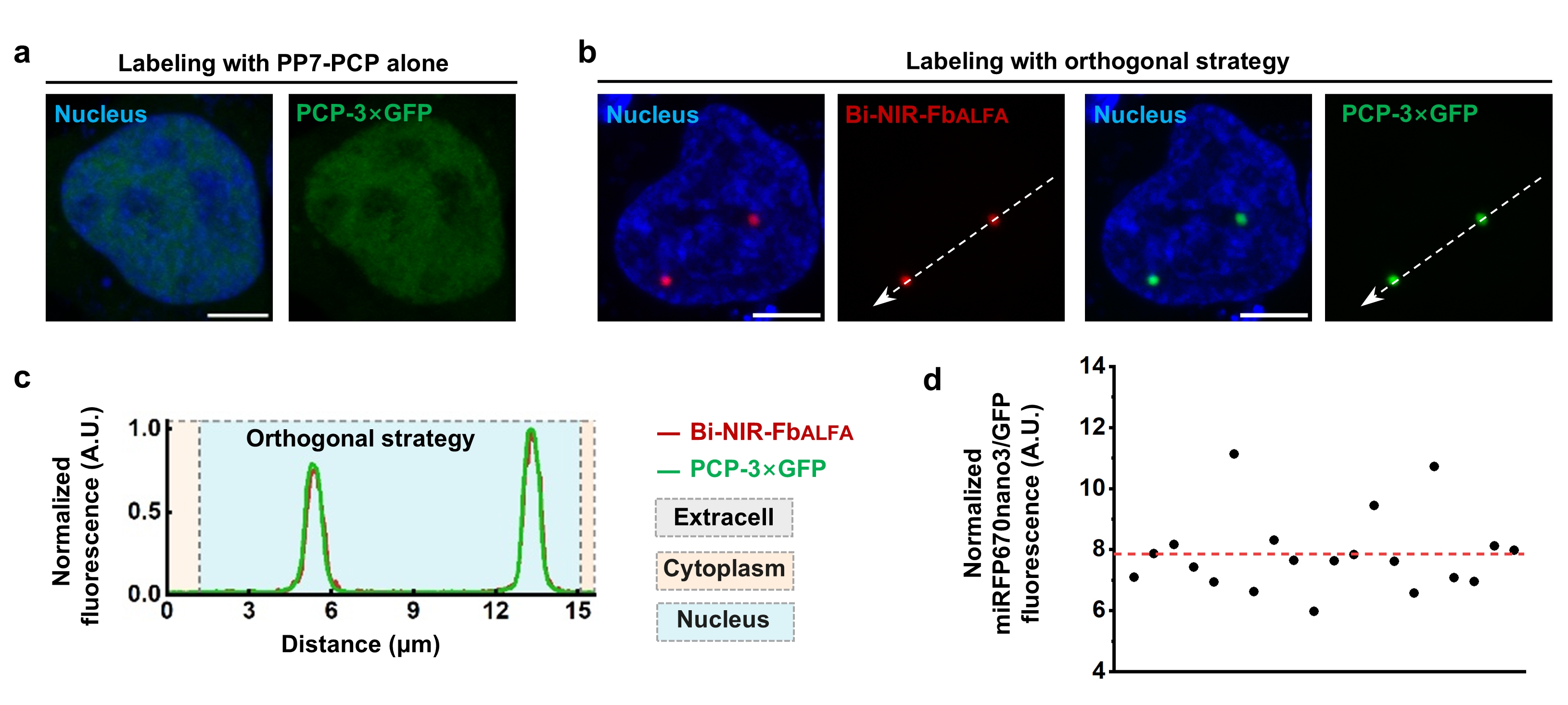


**Figure S13 Bi-NIR-Fb_ALFA_ occupancy on dCas9-16×ALFA revealed from the orthogonal strategy.**

**(a)** Representative images of MUC4.1 labeling with PP7-PCP alone in HEK293T cells. No obvious MUC4.1 puncta were observed due to the limited signal amplification of PP7-PCP alone. Scale bar, 5 μm. **(b)** Representative images of MUC4.1 labeling with orthogonal strategy. Dotted arrow in **(b)** marks regions analyzed by Fiji to produce the fluorescence intensity plot in **(c)**. Scale bar, 5 μm. **(d)** Quantifications of the normalized miRFP670nano3/GFP fluorescence ratio on dCas9-16×ALFA. *n* = 20 puncta. Each dot represents a single punctum and red dashed line indicates the average value. Identical imaging conditions with Figure 2k and Figure S11 were used for the calculation.


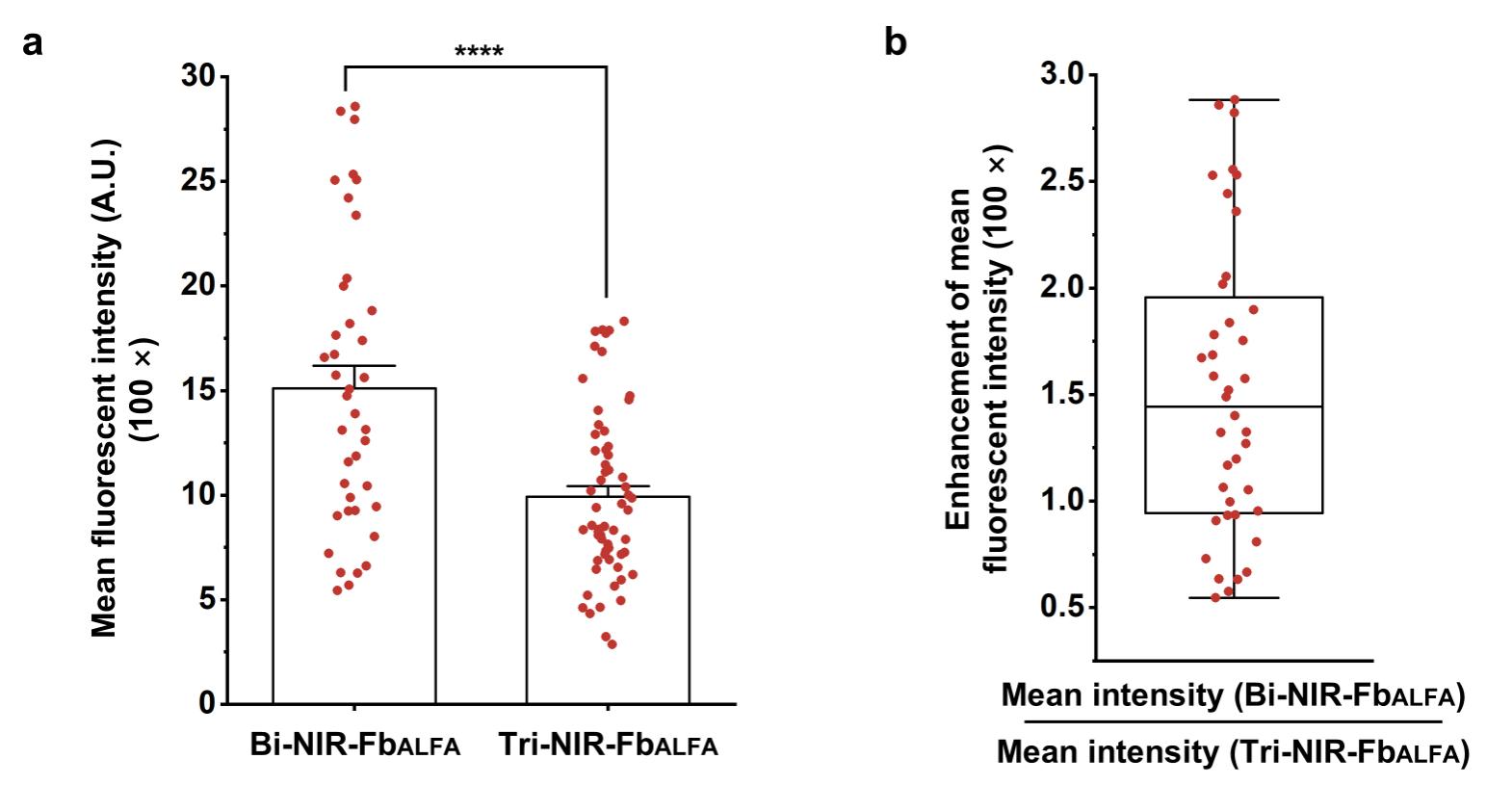


**Figure S14. Bi-NIR-Fb_ALFA_ yielding higher mean fluorescent intensity compared to Tri-NIR-Fb_ALFA_ in MUC4.1 labeling.**

**(a)** Mean fluorescent intensity analysis of MUC4.1 imaging with Bi-NIR-Fb_ALFA_ and Tri-NIR-Fb_ALFA_. Data are presented as means ± SEM for Bi-NIR-Fb_ALFA_ labeling (1510.20 ± 108.50, *n* = 40 puncta) and Tri-NIR-Fb_ALFA_ labeling (991.26 ± 52.09, *n* = 60 puncta). Statistical analysis was performed using the Mann-Whitney test. *p* < 0.0001. **(b)** Enhanced fluorescent intensity of MUC4.1 labeling by Bi-NIR-Fb_ALFA_ compared to Tri-NIR-Fb_ALFA_. Mean ± SD of fluorescent intensity enhancement: 1.52 ± 0.69. Box plots show the median, and the whiskers extend to the minimum and maximum values. Image acquisition was performed under identical conditions for fluorescent intensity analysis.


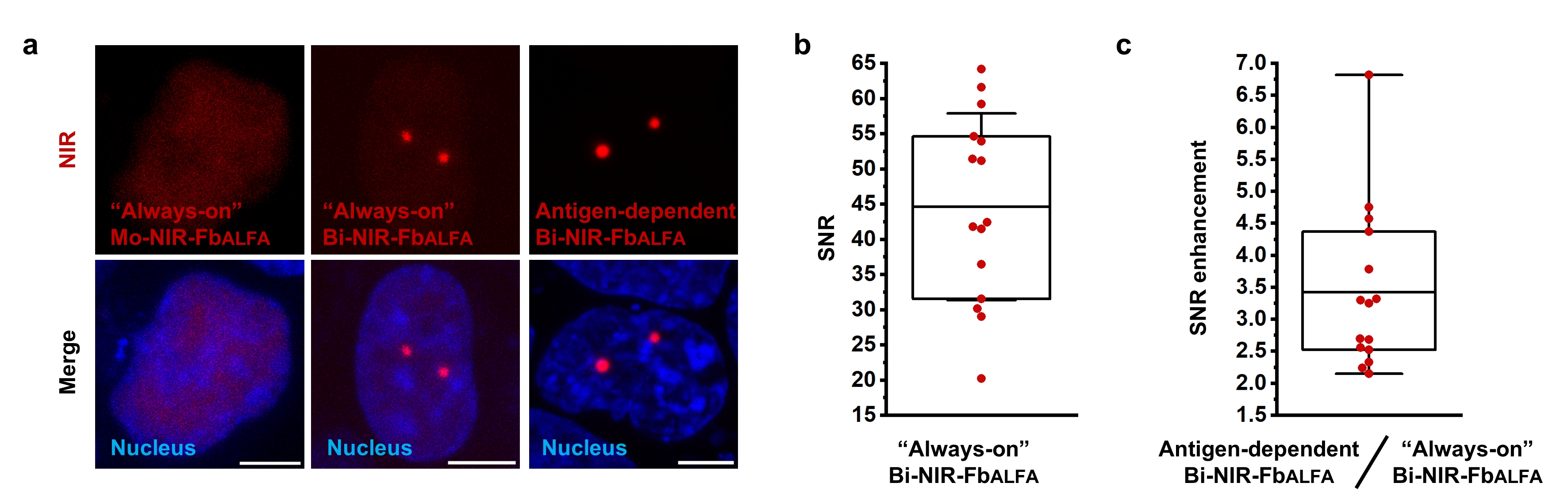


**Figure S15 MUC4.1 labeling under different conditions.**

**(a)** Representative images of MUC4.1 labeled with dCas9-16×ALFA and "always-on" Mo-NIR-Fb_ALFA_, "always-on" Bi-NIR-Fb_ALFA_ and antigen-dependent Bi-NIR-Fb_ALFA_. Scale bar, 5 μm. **(b)** SNR analysis of MUC4.1 labeled with dCas9-16×ALFA and "always-on" Bi-NIR-Fb_ALFA_ in HEK293T cells. Data are presented as means ± SD (44.61 ± 13.27, *n* = 15 puncta). **(c)** SNR enhancement of MUC4.1 labeled with dCas9-16×ALFA and antigen-dependent Bi-NIR-Fb_ALFA_ compared to "always-on" Bi-NIR-Fb_ALFA,_ Mean ± SD of SNR enhancement: 3.42 ± 1.27. Box plots show the median, and the whiskers extend to the minimum and maximum values.


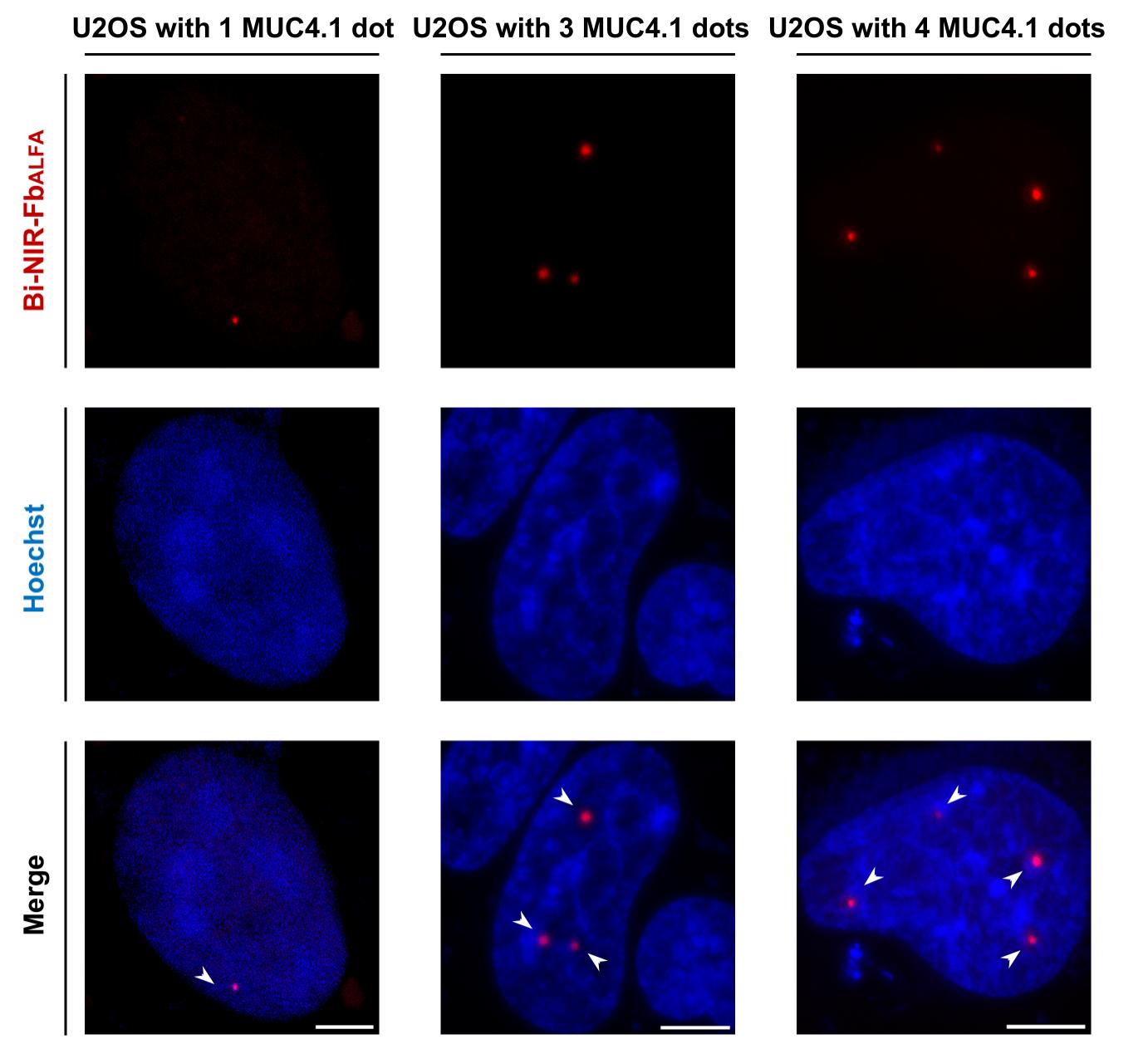


**Figure S16. Heterogeneous MUC4.1 labeling in U2OS cells.**

White arrows indicate BRIGHT dots corresponding to the MUC4.1 locus. Scale bar, 5 μm.


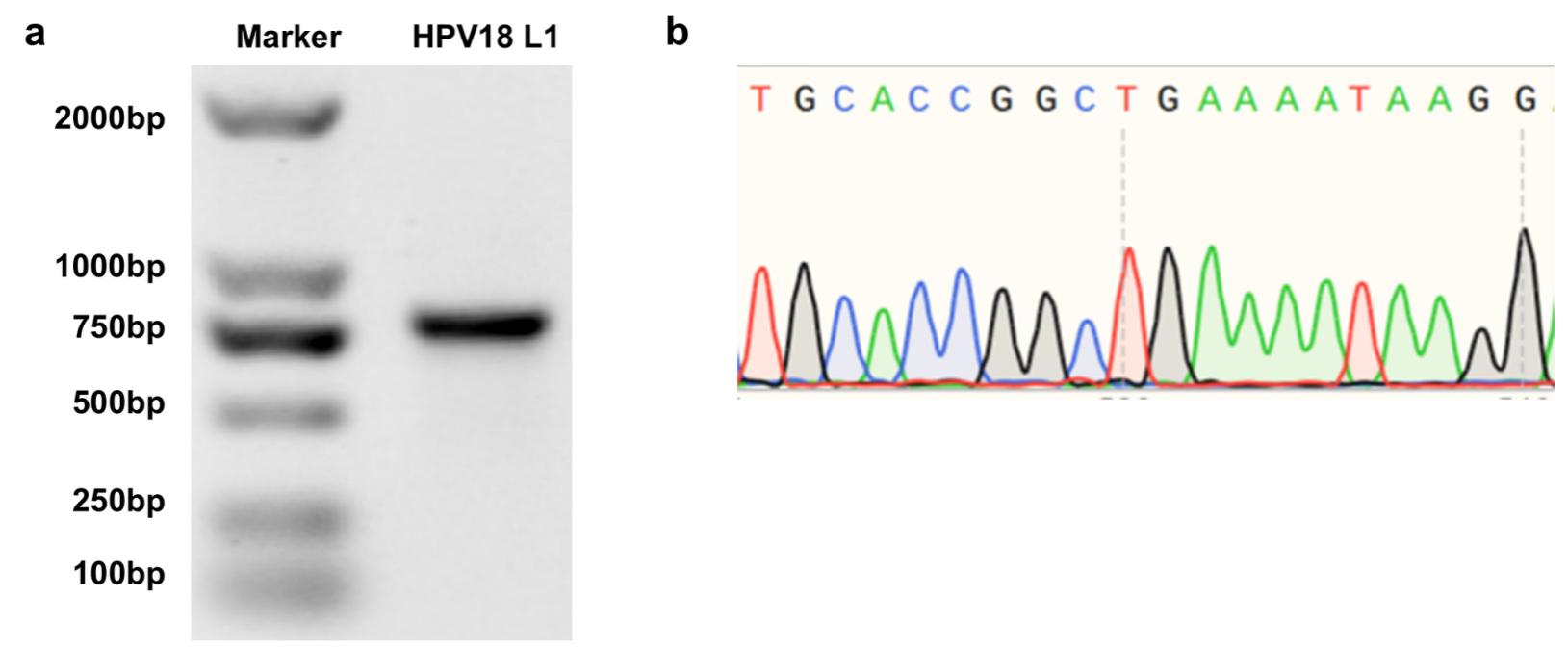


**Figure S17. Verification of the sgRNA target site within the L1 region of HPV-18 integrated in HeLa genome.**

**(a)** Gel electrophoresis analysis of a 769 bp PCR product amplified from the HPV-18 L1 region containing the sgRNA target site, using genomic DNA extracted from HeLa as the template. **(b)** Sanger sequencing chromatogram of the PCR products containing the sgRNA target site.


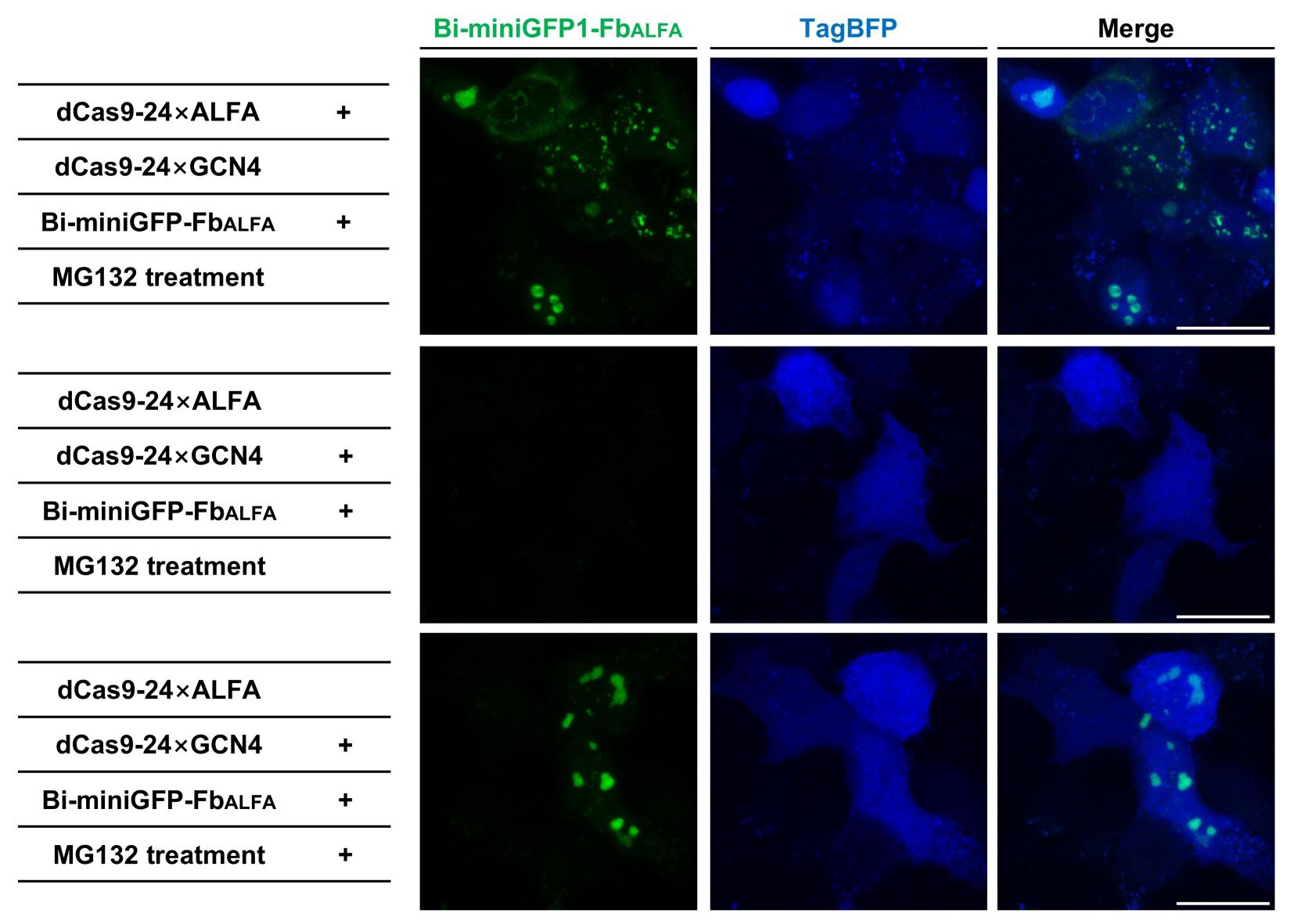


**Figure S18. Bi-miniGFP1-Fb_ALFA_ exhibiting ALFA-dependent fluorescence and proteasomal degradation.**

Representative fluorescence images of the three con-transfection groups. Scale bar, 20 μm.


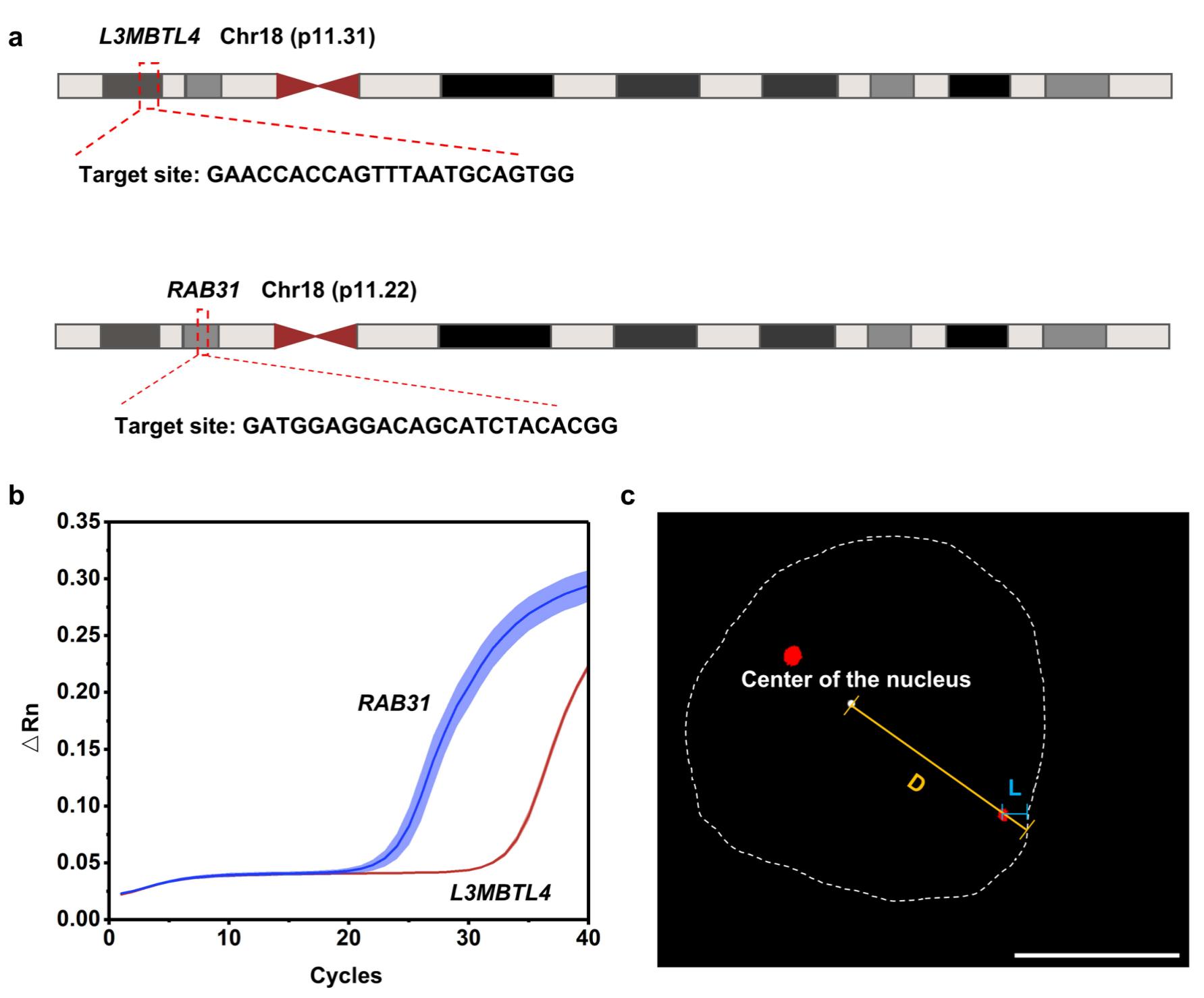


**Figure S19. Characterization of gene location, transcriptional activity, and normalized distance to the nuclear periphery for *L3MBTL4* and *RAB31* genes in HEK293T cells.**

**(a)** Genomic positions and BRIGHT targeting sites of the *L3MBTL4* and the *RAB31* genes. **(b)** RT-qPCR analysis of the expression levels of the *L3MBTL4* gene and the *RAB31* gene. The transcription level of *RAB31* is higher than that of *L3MBTL4*. **(c)** Definition of normalized distance from labeled puncta to the nuclear periphery. The normalized distance is defined as *L*/*D*, where *L* represents the shortest distance from each spot to the nuclear periphery (blue line), and *D* is the distance from the nuclear centroid to the nuclear periphery along the line passing through the spot (yellow line). Scale bar 5um.


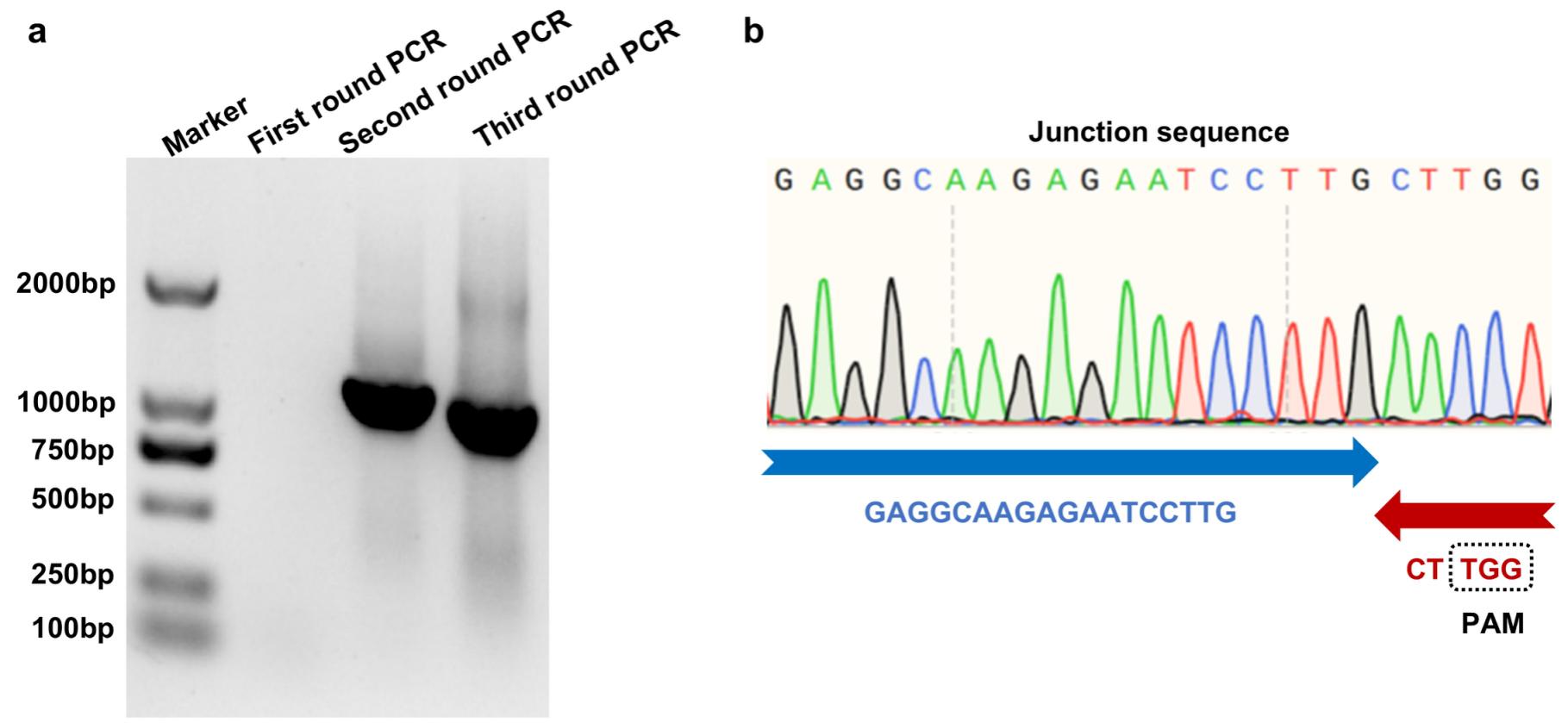


**Figure S20. Verification of the eccBEND3 in HepG2 cells.**

**(a)** Gel electrophoresis analysis of the PCR products obtained from three rounds of amplification of eccBEND3 harboring the junction sequence. **(b)** Sanger sequencing chromatogram of the third-round PCR product containing the junction sequence.

**Table S1. The plasmids used in this study.**

| **Plasmid** | **Vector backbone** | **Description** |
| --- | --- | --- |
| pHRdSV40-NLS-dCas9-1×ALFA-NLS-P2A-TagBFP-dWPRE | pHR | Plasmids expressing dCas9 fused to 1 copy of ALFA peptide under the control of the SV40 promoter. |
| pHRdSV40-NLS-dCas9-4×ALFA-NLS-P2A-TagBFP-dWPRE |  | Plasmids expressing dCas9 fused to 4 copies of ALFA peptide under the control of the SV40 promoter. |
| pHRdSV40-NLS-dCas9-8×ALFA-NLS-P2A-TagBFP-dWPRE |  | Plasmids expressing dCas9 fused to 8 copies of ALFA peptide under the control of the SV40 promoter. |
| pHRdSV40-NLS-dCas9-16×ALFA-NLS-P2A-TagBFP-dWPRE |  | Plasmids expressing dCas9 fused to 16 copies of ALFA peptide under the control of the SV40 promoter. |
| pHRdSV40-NLS-dCas9-24×ALFA-NLS-P2A-TagBFP-dWPRE |  | Plasmids expressing dCas9 fused to 24 copies of ALFA peptide under the control of the SV40 promoter. |
| pcDNA3.1(+)-CMV-Bi-NIR-Fb_ALFA_-NLS | pcDNA3.1(+) | Plasmids expressing Bi-NIR-Fb_ALFA_ under the control of the CMV promoter. |
| pcDNA3.1(+)-EF-1α-Bi-NIR-Fb_ALFA_-NLS |  | Plasmids expressing Bi-NIR-Fb_ALFA_ under the control of the EF-1α promoter. |
| pcDNA3.1(+)-hPGK-Bi-NIR-Fb_ALFA_-NLS |  | Plasmids expressing Bi-NIR-Fb_ALFA_ under the control of the hPGK promoter. |
| pcDNA3.1(+)-minCMV-Bi-NIR-Fb_ALFA_-NLS |  | Plasmids expressing Bi-NIR-Fb_ALFA_ under the control of the miniCMV promoter. |
| pcDNA3.1(+)-hPGK-Bi-NIR-Fb_ALFA_-NLS-always on |  | Plasmids expressing "always-on" Bi-NIR-Fb_ALFA_ under the control of the hPGK promoter. |
| pcDNA3.1(+)-hPGK-Mo-NIR-Fb_ALFA_-NLS |  | Plasmids expressing antigen-dependent Mo-NIR-Fb_ALFA_ under the control of the hPGK promoter. |
| pcDNA3.1(+)-hPGK-Mo-NIR-Fb_ALFA_-NLS-always on |  | Plasmids expressing "always-on" Mo-NIR-Fb_ALFA_ under the control of the hPGK promoter. |
| pcDNA3.1(+)-hPGK-Tri-NIR-Fb_ALFA_-NLS |  | Plasmids expressing Tri-NIR-Fb_ALFA_ under the control of the hPGK promoter. |
| pcDNA3.1(+)-hPGK-Bi-miniGFP-Fb_ALFA_-NLS |  | Plasmids expressing Bi-miniGFP-Fb_ALFA_ under the control of the hPGK promoter. |
| psfGFP-miRFP670nano3-NLS-C1 | pmCherry-C1 | Plasmids expressing GFP-miRFP670nano3 fusion protein. |

The plasmid psfGFP-miRFP670nano3-NLS-C1 is resistant to Kanamycin and other plasmids listed in the table are all resistant to ampicillin.

**Table S2. PCR primers and oligos used in this study.**

| **Name** | **Sequence (5'→3')** |
| --- | --- |
| PCR-F of EF-1α core promoter | ACGACGCGTTGGGCAGAGCGCACATCGCC |
| PCR-R of EF-1α core promoter | GGACGAGCTCCTGTGTTCTGGCGGCAAACCCG |
| PCR-F of hPGK promoter | ACGACGCGTTGGGGTTGGGGTTGCGCCTTTTC |
| PCR-R of hPGK promoter | GGACGAGCTCCTGGGGAGAGAGGTCGGTGATTC |
| Annealing oligo1 for minimal CMV promoter | ACGACGCGTTGGTAGGCGTGTACGGTGGGAGGCCTATATAAGCAGAGCTGAGCTCGTCC |
| Annealing oligo2 for minimal CMV promoter | GGACGAGCTCAGCTCTGCTTATATAGGCCTCCCACCGTACACGCCTACCAACGCGTCGT |
| PCR-F of GFP | CTACCGGTCGCCACCATGGGTGGAGGTGGATCCGGAAGT |
| PCR-R of GFP | AGAGCGGCCGCCAGATTTG |
| PCR-F of miRFP670nano3 | CAAATCTGGCGGCCGCTCTAGCGGTGGAGGAGGTAGCA |
| PCR-R of miRFP670nano3 | CACCTTGCGCTTCTTCTTGGGTTGAACAGACCCCCCGCCA |
| PCR-F of dCas9-24×ALFA and dCas9-24×GCN4 | CCCTGCCCTCCAAATATGTGA |
| PCR-R of dCas9-24×ALFA and dCas9-24×GCN4 | GCACCTTGTCCAGATTAGCG |
| PCR-F of Bi-NIR-Fb_ALFA_ | ATACGTCCACGGCAGATACCAG |
| PCR-R of Bi-NIR-Fb_ALFA_ | CCCCACAGCTTCTTGCCTT |
| PCR-F of β-actin | CACCATTGGCAATGAGCGGTTC |
| PCR-R of β-actin | AGGTCTTTGCGGATGTCCACGT |
| PCR-F of HPV-18 L1 | ACGGCGTGAGCAGCTTTTTG |
| PCR-R of HPV-18 L1 | CCTGGCACGTACACGCACAC |
| PCR-F of *L3MBTL4* | GTAGCTCGGTCGGCGTTG |
| PCR-R of *L3MBTL4* | GCTGAAGGGACGTGACTCAA |
| PCR-F of *RAB31* | ACGGCACATTAGGCAGTTGA |
| PCR-R of *RAB31* | GAGCAGTTTGCACAACAGGG |
| 1^st^ round PCR-F of ecBEND3 | CACTAGTCAGAAATAACTGAAAATCAGACA |
| 1^st^ round PCR-R of ecBEND3 | TCAGTATCAGGGCATTGATTGCTCAATTGA |
| 2^nd^ round PCR-F of ecBEND3 | ATTAAGAGGGTTGATTGATTGATTGATTTG |
| 2^nd^ round PCR-R of ecBEND3 | ATAAGTCATTTACTGGCCTTTTCTTGTATG |
| 3^rd^ round PCR-F of ecBEND3 | AGATGGAGTCTCGCTCTGTTGCCCAGGATG |
| 3^rd^ round PCR-R of ecBEND3 | TCAGGGGCTGTTTTGGTTTGAGCATGGAGC |

**Table S3. The spacer of sgRNA used in this study.**

| **Spacer of sgRNA** | | **Chromatin context** | **Sequence information** | | | | |
| --- | --- | --- | --- | --- | --- | --- | --- |
|  |  |  | **Start** | **End** | **Spacer** | **PAM** | **Copies** |
| High copy number | Telomere | LADs in heterochromatic regions | - | - | TAGGGTTAGGGTTAGGGTTA | GGG | - |
|  | Chr3Rep |  | 195199025 | 195233876 | TCCTCTGTATGATATCACAG | TGG | 333 |
|  | Chr13Rep |  | 112930813 | 112973591 | GGTAAGCATGGACCATTCCTTC | AGG | 350 |
|  | Chr3-M4-E2Rep | Non-LADs in euchromatic regions | 195505721 | 195515533 | GTGGCGTGACCTGTGGATGCTG | AGG | 100-400 |
| Low copy number | Chr21Rep | LADs in heterochromatic regions | 44007543 | 44010498 | GGTAAGGGGAAACGCCTGCG | TGG | 54 |
|  | Chr7-*DPP6* |  | 154660290 | 154664271 | GGTGAATCACCATGGCGTAT | TGG | 38 |
|  | Chr19Rep |  | 403972 | 405094 | GGACGGGGTCCTGCCCTGAG | GGG | 30 |
|  | Chr19-*TDRD12* |  | 32745723 | 32745742 | GGAGAGAGACTGGCTGATG | TGG | 21 |
|  | Chr10-*DOCK1* |  | 127396902 | 127397642 | GATCTGAGCATGAGTTACAC | GGG | 13 |
|  | Chr17-*LINC20887* | Non-LADs in euchromatic regions | 66744 | 68352 | GTAATAACTGCCCCAGA | TGG | 14 |
|  | Chr9-*PNPLA7* |  | 140427333 | 140428887 | ATTCTGGGAGTCCTCCCGCC | TGG | 9 |
|  | Chr13-*POLR1D* |  | 27623063 | 27623225 | GAAGTGGGAATGAGTTGGAGG | TGG | 5 |
|  | ChrX-*XIST* |  | 73844748 | 73846233 | GACCCCTTTTGCAGTACAGC | AGG | 3 |
| Single copy number | Chr3-MUC4.1 | Non-LADs in euchromatic regions | 195535377 | 195535397 | GTAAAGTAGAAAAGGCATAAA | GGG | 1 |
|  | Chr3-MUC4.2 |  | 19553832 | 195538353 | GAAGAGTGGAGGCCGTGCGCGG | TGG | 1 |
|  | Chr3-MUC4.3 |  | 195537581 | 195537605 | GCAAGCAAGGGAAGCGACAAGG | AGG | 1 |
|  | Chr3-MUC4.4 |  | 195537748 | 195537772 | GTACACCCTTGTGTACAGAGCT | GGG | 1 |
|  | Chr3-*PPP1R2* |  | 195247315 | 195247337 | GAGGACTGGCTAAGCTTAGA | AGG | 1 |
|  | Chr13-*SOX1* | LADs in heterochromatic regions | 112722511 | 112722534 | GCTACGCGCACGTCAACGGCT | GGG | 1 |
| Other spacers | HPV-18 | - | - | - | TGCTGCACCGGCTGAAAATA | AGG | 1 |
|  | Chr18-*L3MBTL4* | LAD site | 6272376 | 6273541 | GAACCACCAGTTTAATGCAG | TGG | 10 |
|  | Chr18-*RAB31* | Non-LAD site | 9815244 | 9816153 | GATGGAGGACAGCATCTACA | CGG | 20 |
|  | eccBEND3 | - | - | - | GAGGCAAGAGAATCCTTGCT | TGG | 1 |

**References:**

[1] J. Abramson, J. Adler, J. Dunger, et al., "Accurate structure prediction of biomolecular interactions with AlphaFold 3" *Nature* (2024): 493, https://doi.org/10.1038/s41586-024-07487-w.

[2] E. F. Pettersen, T. D. Goddard, C. C. Huang, et al., "UCSF ChimeraX: Structure visualization for researchers, educators, and developers" *Protein Sci* (2021): 70, https://doi.org/10.1002/pro.3943.

[3] M. E. Tanenbaum, L. A. Gilbert, L. S. Qi, J. S. Weissman, R. D. Vale, "A protein-tagging system for signal amplification in gene expression and fluorescence imaging" *Cell* (2014): 635, https://doi.org/10.1016/j.cell.2014.09.039.
