# Supplementary material for: BRIGHT Enables High‐SNR Live‐Cell Imaging of Non‐Repetitive Sequences via Bivalent Fluorescent Nanobody‐Mediated Cascade‐Dependent Illumination
Source: Adv Sci (Weinh). 2025 Oct 17;13(2):e13014. doi: 10.1002/advs.202513014 (PMC12786294; doi:10.1002/advs.202513014)
Supplement: Supplementary file 2 — Supporting Information [file ADVS-13-e13014-s002.docx]

**Video Legends**

**Video S1.** **Time-lapse imaging of Chr3Rep dynamics tracked by BRIGHT in a representative HEK293T cell.**

Chr3Rep loci are labeled by BRIGHT (red). Images were taken with a Multi-SIM (Naxin Optoelectronics Co., Ltd., Beijing, China), spaced by 200ms with a total of 50 shots. Scale bar, 2 μm.

**Video S2.** **Time-lapse imaging of Chr3Rep dynamics tracked by SunTag in a representative HEK293T cell.**

Chr3Rep loci are labeled by SunTag (green). Images were taken with a Multi-SIM (Naxin Optoelectronics Co., Ltd., Beijing, China), spaced by 200ms with a total of 50 shots. Scale bar, 2 μm.

**Video S3.** **Time-lapse imaging of Chr3Rep dynamics tracked by PP7-PCP in a representative HEK293T cell.**

Chr3Rep loci are labeled labeled by PP7-PCP (green). Images were taken with a Multi-SIM (Naxin Optoelectronics Co., Ltd., Beijing, China), spaced by 200ms with a total of 50 shots. Scale bar, 2 μm.

**Video S4.** **Time-lapse imaging of *L3MBTL4* dynamics tracked by BRIGHT in a representative HEK293T cell.**

*L3MBTL4* loci are labeled by BRIGHT (red). Images were taken with a Multi-SIM (Naxin Optoelectronics Co., Ltd., Beijing, China), spaced by 200ms with a total of 50 shots. Scale bar, 2 μm.

**Video S5.** **Time-lapse imaging of *RAB31* dynamics tracked by BRIGHT in a representative HEK293T cell.**

*RAB31* loci are labeled by BRIGHT (red). Images were taken with a Multi-SIM (Naxin Optoelectronics Co., Ltd., Beijing, China), spaced by 200ms with a total of 50 shots. Scale bar, 2 μm.

**Video S6.** **Time-lapse imaging of eccBEND3 dynamics tracked by BRIGHT in a representative HepG2 cell.**

Copies of eccBEND3 are labeled by BRIGHT (red). Images were taken with a Multi-SIM (Naxin Optoelectronics Co., Ltd., Beijing, China), spaced by 200ms with a total of 50 shots. Scale bar, 2 μm.
